# Six Drivers of Aging Identified Among Genes Differentially Expressed With Age

**DOI:** 10.1111/acel.70225

**Published:** 2025-10-13

**Authors:** Ariella Coler‐Reilly, Zachary Pincus, Erica L. Scheller, Roberto Civitelli

**Affiliations:** ^1^ Division of Bone and Mineral Diseases, Musculoskeletal Research Center Washington University School of Medicine St. Louis Missouri USA; ^2^ Department of Medicine Washington University School of Medicine St. Louis Missouri USA; ^3^ Department of Medical Scientist Training Program Washington University School of Medicine St. Louis Missouri USA; ^4^ Department of Developmental Biology Washington University School of Medicine St. Louis Missouri USA; ^5^ Department of Genetics Washington University School of Medicine St. Louis Missouri USA; ^6^ Department of Cell Biology and Physiology Washington University School of Medicine St. Louis Missouri USA

## Abstract

Many studies have compared gene expression in young and old samples to gain insights on aging, the primary risk factor for most chronic diseases. However, these studies only identify associations without distinguishing drivers of aging from compensatory geroprotective responses or incidental downstream effects. Here, we introduce a workflow to characterize causal effects of differentially expressed genes on lifespan. First, we performed a meta‐analysis of 25 gene expression datasets comprising samples of various tissues from healthy, untreated adult mammals (humans, dogs, and rodents) at two distinct ages. Genes were ranked by the number of datasets in which they exhibited consistent differential expression with age. The top age‐upregulated genes were TMEM176A, EFEMP1, CP, and HLA‐A; the top age‐downregulated genes were CA4, SIAH, SPARC, and UQCR10. Second, the effects of the top ranked genes on lifespan were measured by applying post‐developmental RNA interference of the corresponding ortholog in *Caenorhabditis elegans* . Out of 10 age‐upregulated and 9 age‐downregulated genes that were tested, two age‐upregulated genes (*csp‐3*/CASP1 and *spch‐2*/RSRC1) and four age‐downregulated genes (*C42C1.8*/DIRC2, *ost‐1*/SPARC, *fzy‐1*/CDC20, and *cah‐3*/CA4) produced significant and reproducible lifespan extension. Notably, the data do not suggest that the direction of differential expression with age is predictive of the effect on lifespan. Our study provides novel insight into the relationship between differential gene expression and aging phenotypes, pilots an unbiased workflow that can be easily repeated and expanded, and pinpoints six genes with evolutionarily conserved, causal roles in the aging process for further study.

## Graphical abstract

A meta‐analysis of 25 transcriptomic datasets from mammalian tissues revealed 45 genes consistently upregulated or downregulated with age. Orthologs of these genes were functionally evaluated using *Caenorhabditis elegans* lifespan screens to reveal those with causal roles in the aging process, thus identifying six evolutionarily conserved drivers of aging.

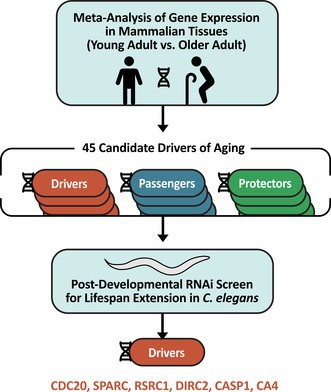

## 1 Introduction

Advanced age is the primary risk factor for most chronic diseases, and as our population ages, the social and economic burden of chronic disease continues to grow year by year (Kennedy et al. 2014). The field of geroscience has emerged to study the mechanisms underlying aging itself and develop strategies to combat age‐related decline, or senescence, at the source. According to the widely accepted evolutionary theory of aging, senescence is pervasive because there is negligible selection pressure during the post‐reproductive period, a phenomenon known as the “selection shadow” (Kirkwood and Austad 2000; Austad and Kirkwood 2008). It is therefore crucial to study the role of genetic variants and gene expression changes in aging in order to uncover potentially advantageous adjustments that have been masked by the selection shadow.

Specialized approaches are needed to detect age‐related gene expression signals, which are often subtle and widespread rather than striking and targeted. Indeed, as noted in the *Handbook of the Biology of Aging*, differentially expressed genes (DEGs) with the largest fold changes are frequently found to be downstream targets rather than upstream regulators (Hou et al. 2016). Moreover, age‐related phenomena such as transcriptional drift create reproducible expression patterns that are nonetheless stochastic and unregulated, further obscuring meaningful signals (Perez‐Gomez et al. 2020; Bahar et al. 2006; Rangaraju et al. 2015). As stochastic signals are unlikely to replicate across species and tissues, and since frequency is more meaningful than fold change, drivers of aging may ideally be identified using a multi‐species, multi‐tissue meta‐analysis using the value‐counting method. This strategy, first pioneered by de Magalhães et al. (2009) and further developed by Palmer et al. (2021), has been used to catalog numerous individual DEGs as well as broader patterns in functional enrichment and pathway analysis. However, translation of such findings into actionable therapeutic strategies is challenging. Any upregulated gene presumed to be a driver of aging could just as easily be a compensatory geroprotective response or an unimportant downstream effect, often called a “passenger” to contrast with the aforementioned “driver” (Perez‐Gomez et al. 2020; de Magalhães and Toussaint 2004). In other words, as the age‐old adage warns, correlation does not necessarily equal causation.

Functionally evaluating genes related to aging also presents special challenges. Stable cell lines cannot be used to study aging in vitro because of the immortal nature of such lines. In vivo models are more useful, but require time and resources to age the animals and monitor them until their natural death. In mammals, this can entail years of labor, and this is surely one reason why the short‐lived nematode *Caenorhabditis elegans* has been such a popular model organism in geroscience for decades (Johnson 2008). Although nematodes are only distant relatives of humans, they share remarkably similar features of post‐reproductive senescence, including sarcopenia and reduced motility, deteriorated learning and memory, and weakened immunity (Wilkinson et al. 2012; Son et al. 2019; Chen et al. 2013). In contrast to humans or any mammal, these age‐dependent changes occur on a compressed timescale of days rather than years, with an average lifespan of only a few weeks. On a genetic level, orthologs of roughly half of all human genes have been identified, and tools have been developed to rapidly, easily, and inexpensively knock down those genes in *C. elegans* worms, making them an ideal choice for reverse genetic screens (Sutphin and Korstanje 2016). However, it is difficult to substantiate findings in *C. elegans* as relevant to human physiology without any means of contextualizing the results in mammalian systems.

Here, we introduce a workflow to unify two separate approaches, analysis of mammalian DEGs and genetic screening in *C. elegans* , leveraging the strengths of each to mitigate their respective weaknesses. We first performed a meta‐analysis comparing gene expression in young adults vs. older adults using publicly available datasets comprising samples of various tissues from healthy, untreated mammals (humans, dogs, and rodents). DEGs were ranked by the consistency of differential expression with age across the largest number of datasets. The highest ranking DEGs with known orthologs in *C. elegans* were then tested using post‐developmental RNA inactivation (RNAi) lifespan assays. Ultimately, we identified six genes with evolutionarily conserved, causal effects on aging that may be prioritized for future mechanistic studies. In addition, we have established a proof of principle for a unified approach for studying evolutionarily conserved mechanisms of aging.

## 2 Material and Methods

### 2.1 Meta‐Analysis Dataset Selection

This meta‐analysis was designed as a simple and scalable approach that is nonetheless highly capable of identifying a collection of genes consistently associated with mammalian aging. The intention was to extricate subtle but meaningful age‐related signals from a background of transcriptional drift and stochastic changes that are unlikely to replicate across species, tissues, and experimental platforms. Thus, as the inclusion criteria and exclusion criteria detailed in Table 1 show, any datasets comprising samples from mammals representative of typical individuals at both young adult and older adult time points were eligible for inclusion.

**TABLE 1:** Inclusion and exclusion criteria for the meta‐analysis of genes differentially expressed during mammalian aging.

Criteria	Included	Excluded
Data availability	Stored in NCBI GEO database, Includes “age” as subset variable	All other datasets
Species	Mammal (human, mouse, rat, dog)	Non‐Mammal (e.g., drosophila, *C. elegans* …)
Tissue type	All (heart, muscle, brain, liver, fat, immune cells…)	None
Age	Young adult and older adult in distinct groups	Early development, including embryonic stages as well as juveniles
Condition	Healthy, untreated	All diseases (e.g., lupus, tumor samples), All interventions including drugs as well as lifestyle interventions like diet and exercise
Genotype	Wild‐type, No genetic condition specified	Mutant, transgenic animals, Humans with specified genetic conditions
Sample size	Any (no specific minimum)	Datasets with low sample size became naturally excluded when they yielded no DEGs

Abbreviations: DEGs, differentially expressed genes; NCBI GEO, National Center for Biotechnology Information, Gene Expression Omnibus.

Gene expression data were obtained from publicly available datasets hosted on the National Center for Biotechnology Information (NCBI) Gene Expression Omnibus (GEO) repository (Edgar et al. 2002). The filters “organism: mammal” and “subset variable type: age” were used to identify roughly 200 curated datasets as candidates for the present study as of March 2021. These datasets were then manually inspected using the inclusion and exclusion criteria (Table 1) to identify 25 suitable datasets, which are listed in Table S1.

### 2.2 Identification of Differentially Expressed Genes (DEGs)

The analysis of gene expression data was conducted using the R software environment version 3.2.3 (R Core Team 2015) and a series of packages from the Bioconductor project, including *GEOquery* version 2.40.0 (Davis and Meltzer 2007), *limma* version 3.26.8 (Ritchie et al. 2015), and *BioBase* version 2.30.0 (Huber et al. 2015). Briefly, by adapting scripts from GEO's own GEO2R tool (Barrett et al. 2013), the data was retrieved and translated to R‐compatible formats via *GEOquery* and analyzed for DEGs via *limma*. DEGs were calculated by comparing samples from young vs. old tissues for each individual dataset; individual samples were included or excluded from the analysis using the aforementioned inclusion and exclusion criteria (Table 1). To cast a wide net with high sensitivity to detect DEGs even in datasets with small sample sizes, candidate DEGs were identified in individual datasets using the permissive threshold of adjusted *p* value < 0.25, controlling for false discovery rates (FDR) using the Benjamini‐Hochberg method. Finally, to facilitate analysis across datasets, all DEGs from nonhuman datasets were converted to their human homologs using the *homologene* package version 1.4.68.19.3.27 (Mancarci, n.d.).

### 2.3 Value‐Counting Method for Ranking DEGs

The genes were then scored using a variation of the value‐counting method first established in the cancer field (Rhodes et al. 2004) and later applied to age‐dependent gene expression (de Magalhães et al. 2009). This approach enables the integration of gene expression data from diverse species, tissues, platforms, and experimental designs while remaining highly scalable and reproducible. In brief, genes are ranked by the number of datasets in which they are identified as a DEG according to a chosen threshold. Thus, the consistency of differential expression across a variety of datasets is prioritized, whereas individual effect sizes are discarded.

Here, a new variation of the value‐counting method was introduced to further prioritize consistency: ranking was determined based on the absolute value of the difference between upregulation and downregulation scores, where scores were determined by the number of datasets in which the DEG was significantly upregulated and downregulated with age, respectively. This is written formulaically below along with an example.

Let scores $S_{i_{\mathrm{Up}}}$ and $S_{i_{\text{Down}}}$ represent the number of datasets in which gene i is significantly upregulated and downregulated with age, respectively. The total score $S_{i}$ and rank $R_{i}$ for each gene i is calculated as follows:

$$S_{i}=S_{i_{\mathrm{Up}}}-S_{i_{\text{Down}}}$$

$$R_{i}=\left|S_{i}\right|=\left|S_{i_{\mathrm{Up}}}-S_{i_{\text{Down}}}\right|$$

For example, if a gene was significantly (adj. *p* < 0.25) upregulated in 2 datasets and downregulated in 8 datasets out of the total 25 datasets analyzed, this gene would have a rank of $\left|2-8\right|=6$. It is important to highlight that a gene with a total score and rank of 0 does not necessarily indicate that the gene was differentially expressed in none of the datasets, as it could also be upregulated and downregulated in an equal number of datasets.

Based on the average number of DEGs identified per dataset in the previous step being 1816 genes out of an average over 30,000 probes per dataset, a binomial distribution with a success rate of 6% and 25 trials can be applied to estimate the final *p* value for high‐ranking genes. For DEGs of rank 6 or above, the cumulative probability $P\left(X\geq 6\right)$ yields a final *p* value of 0.003:

PX≥6=∑j=6n25j0.06j1−0.0625−j

DEGs with a rank of at least 7 ($R_{i}\geq 7$) were further analyzed for gene expression patterns across tissues by normalizing to the number of datasets from each tissue type that were analyzed.

This value‐counting analysis was conducted using the python software environment version 3.11.5 (van Rossum and Drake Jr 1995), and the data were visualized utilizing the *pandas*, *matplotlib*, and *seaborn* packages (McKinney 2010; Hunter 2007; Waskom 2021).

### 2.4 Pathway Analysis

DEGs with a rank of at least 6 ($R_{i}\geq 6$) were analyzed using pathway analysis in the R software environment to explore their known roles in key biological processes. Rank 6 was set as the cutoff to ensure that the gene would have < 1% chance of achieving this rank by chance alone (*p* = 0.003, per the binomial distribution above). The Bioconductor package *clusterProfiler* version 4.8.0 (Wu et al. 2021) was used to perform gene ontology (GO) enrichment analysis. DEGs were mapped to GO biological processes, cellular components, and molecular functions using standard settings (Benjamini‐Hochberg adjusted *p* < 0.05).

### 2.5 Identifying Worm Orthologs of DEGs

*C. elegans* orthologs of DEGs with a rank of at least 7 ($R_{i}\geq 7$) were identified using OrthoList 2, which is a compendium of worm genes with human orthologs compiled by a meta‐analysis of several orthology prediction methods (Kim et al. 2018). Where multiple orthologs were available for a given DEG, the highest confidence ortholog was chosen, as indicated by the number of orthology prediction methods supporting orthology. Where multiple orthologs and/or clones were available for a given gene without any discernible way to prioritize one over another, the first item listed in the results was chosen. The final list of orthologs along with the availability of corresponding RNAi clones is shown in Tables S2 and S3. In some cases, when a clone could not be cultured or verified by sequencing (as outlined in the next section below), experiments were conducted using the next clone on the list.

### 2.6 Worm Culture and Post‐Developmental RNAi

Wild‐type (N2) *C. elegans* worms were maintained on plates of solid nematode growth media (NGM) seeded with *Escherichia coli* OP50 bacteria at 20°C using standard protocols (Sutphin and Kaeberlein 2009). *E. coli* HT115 bacteria clones carrying RNAi constructs of interest were obtained from the Ahringer RNAi library (Kamath and Ahringer 2003) and seeded onto solid NGM plates containing Isopropyl β‐D‐1‐thiogalatopyranoside (IPTG) and ampicillin according to standard protocols for the RNAi feeding method (Wilkinson et al. 2012; Timmons and Fire 1998). Briefly, for each gene of interest, an individual colony of RNAi bacteria was cultured in liquid LB medium overnight and then seeded onto plates the following day. In parallel, to confirm the identity of the clones, DNA was isolated from the same culture using the QIAprep Spin Miniprep Kit (QIAGEN, Hilden, Germany), and the inserts were sequenced with an M13‐forward primer using standard Sanger sequencing services by Azenta Life Sciences (South Plainfield, NJ, USA). The seeded plates were incubated at room temperature for 2–3 days, during which time 2′ fluro‐5′ deoxyuridine (FUDR) was added to the plates 24–48 h before transferring worms. Worms were age‐synchronized using the bleaching method with L1 synchronization and allowed to develop to the late L4 stage on standard OP50 plates before being transferred to the plates seeded with the RNAi feeding bacteria, as described in previous post‐developmental RNAi screens (Curran and Ruvkun 2007; Chen et al. 2007).

### 2.7 Lifespan Extension Screen

Lifespan assays were conducted using standard protocols (Sutphin and Kaeberlein 2009). Briefly, worms were scored as alive or dead every 2–3 days by visual observation: apparently motionless worms were gently prodded with a platinum wire pick, and worms that failed to react were scored as dead and removed from the plate. Worms that left the plate surface and dried on the plate wall were censored. For the initial screening, the 19 candidate clones were tested against within‐batch GFP RNAi negative controls as well as the well‐known *daf‐2* RNAi positive control (Dillin et al. 2002). For every clone tested, the initial screening included roughly 80–100 worms spread across multiple plates, with approximately 20 worms per plate. For the subsequent validation of the clones that significantly extended lifespan in the initial screening, each group included roughly 100–120 worms, with approximately 25 worms per plate, tested against GFR RNAi and empty L4440 vector negative controls as well as the *daf‐2* RNAi positive control.

### 2.8 Lifespan Extension Analysis

Lifespan was defined as the number of days until death, starting from the first day of adulthood (3 days after L1 synchronization). The Online Application for Survival Analysis 2 (OASIS 2) tool was used to calculate mean, median, and maximum lifespans for each group as well as to compare test groups using the log‐rank test (Han et al. 2016). An RNAi clone was considered to have extended lifespan if the log‐rank test comparing that clone to the GFP RNAi negative control within the same batch was significant (*p* < 0.05 with Bonferroni multiple test correction) in both the initial screen and the subsequent validation screen. Survival data was then plotted as survival curves using GraphPad Prism version 10.2.3.403 for Windows.

## 3 Results

### 3.1 Meta‐Analysis Datasets Were Derived From a Variety of Mammalian Tissues

Twenty‐five publicly available gene expression datasets were selected from the NCBI GEO repository according to the inclusion and exclusion criteria outlined in Table 1, and their traits and NCBI identification numbers are listed in Table S1. The predominant species represented in this analysis was mouse, constituting roughly half the datasets (13), followed by human (6), then rat (5), then dog (1). Most datasets were derived from muscle (7) and brain (5) tissues, but also well represented were adipose tissues (3) as well as immune cells and their precursors (3), with smaller contributions from the heart, liver, trachea, cochlea, and reproductive tissues (Figure S1A). The number of DEGs extracted from each dataset varied widely, ranging from six genes to 3631 genes (median, 1509; interquartile range 466–3159). If each instance of a DEG being extracted is considered a data point, then the sum total of data points contributed by most tissues ranged from roughly 4000 to 11,000; however, cochlea, trachea, and reproductive tissues contributed strikingly fewer, with less than 1000 data points each (Figure S1B). These results reflect the wide variety of studies contributing to this analysis, with varying experimental methods as well as unequal availability of samples from different tissues, particularly from human subjects.

### 3.2 High‐Ranking Genes Were Consistently Differentially Expressed With Age Across Diverse Tissues

**FIGURE 1:**
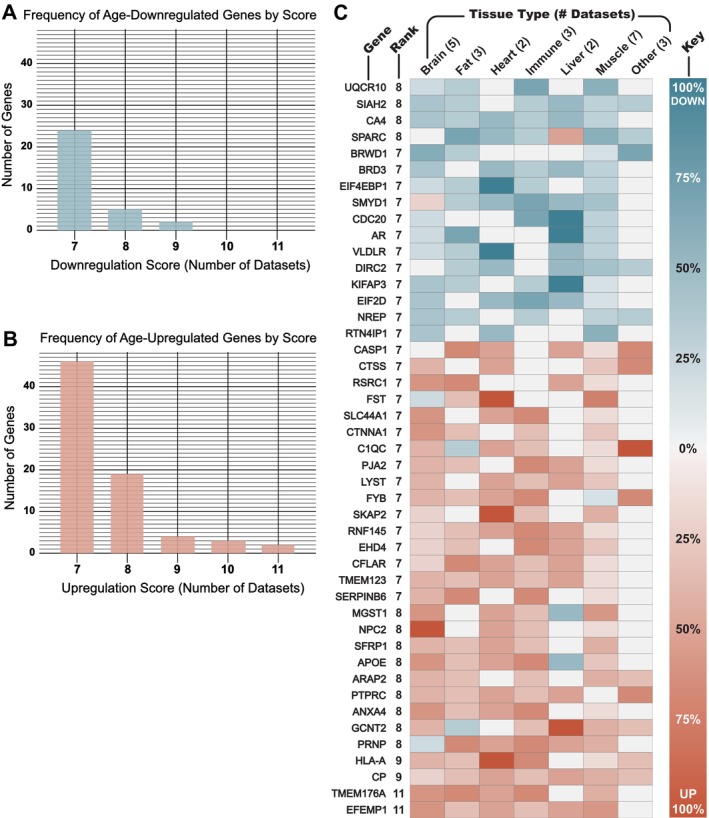
Genes most consistently differentially expressed with age in mammalian tissues. Every gene had a downregulation score $S_{\text{Down}}$ and an upregulation score $S_{\mathrm{Up}}$ representing the number of datasets in which the gene was significantly downregulated and upregulated with age, respectively (Benjamini‐Hochberg adjusted *p* < 0.25). The rank R of each gene was calculated as the absolute value of the difference between these two scores, the total score S: $R=\left|S\right|=|S_{\mathrm{Up}}-S_{\text{Down}}|$. (A) There were 31 genes with $S_{\text{Down}}\geq 7$, and the most consistently downregulated genes had a score of $S_{\text{Down}}=9$. (B) There were 74 genes with $S_{\mathrm{Up}}\geq 7$, and the most consistently upregulated genes had a score of $S_{\mathrm{Up}}=11$. (C) The heatmap shows the gene symbol, rank R, and normalized tissue‐specific expression trends for all 45 genes of rank R≥7, comprising 29 age‐upregulated genes $\left(S>0\right)$ and 16 age‐downregulated genes $\left(S<0\right)$. Heatmap values range from 100% down to 100% up, representing as a percentage the fraction $|S_{\text{tissue}}/n_{\text{tissue}}|$, or the total score S divided by the number of datasets n derived from only the specified tissue type.

Using the value‐counting method, every gene was assigned upregulation and downregulation scores corresponding to the number of datasets in which that gene was significantly upregulated and downregulated with age, respectively (FDR‐adjusted *p* < 0.25). In general, more genes were commonly upregulated than downregulated with age. Out of a highest possible score of 25 (total number of datasets), the highest downregulation score was 9 (Figure 1A), and the highest upregulation score was 11 (Figure 1B). Similarly, only 31 genes achieved a downregulation score of 7 or more, whereas 74 genes reached an upregulation score of 7 or more.

To narrow the list of DEGs to those with the most consistent age‐related trends, genes were ranked according to the absolute value of the difference between their upregulation and downregulation scores. Thus, genes exhibiting opposing trends in different species or tissues were not ranked highly. Although there were 105 genes with a downregulation or upregulation score of at least 7, there were only 45 genes that ranked 7 or above after the opposing score was subtracted. The highest‐ranking age‐upregulated genes were EFEMP1 (Rank 11), TMEM176A (11), CP (9), and HLA‐A (9); the highest‐ranking age‐downregulated genes were CA4 (8), SIAH2 (8), SPARC (8), and UQCR10 (8). Ranks were used to select DEGs for further analyses and experiments: rank 6 was used as the cut‐off to select 130 DEGs for pathway analysis, and rank 7 was used as the cutoff to select 45 DEGs for in vivo testing in *C. elegans* . Although rank 6 was statistically sufficient per the binomial distribution described in the Methods section, the resource‐intensive nature of the in vivo experiments necessitated further narrowing of the list to fewer candidates with the most consistent gene expression patterns.

The 45 highest‐ranking DEGs, comprising 16 age‐downregulated and 29 age‐upregulated genes, are listed in Figure 1C with a heatmap displaying the tissues that contributed to each gene's rank. For example, EFEMP1, which was tied for the highest‐ranking gene, was significantly upregulated in datasets from mouse liver and hematopoietic stem cells, rat heart and adipose tissues, and both human and mouse brain and muscle tissues; EFEMP1 was not significantly downregulated in any of the 25 datasets analyzed. As illustrated in the heatmap, no gene was able to achieve this high rank without being consistently differentially expressed in datasets from at least three distinct tissue types, and often more. The genes CA4 and CP were notable for being consistently differentially expressed across all six major tissue types studied as well as being among the top four highest‐ranking downregulated and upregulated genes, respectively. The only gene differentially expressed in 100% of datasets from a major tissue type (3 or more datasets) was NPC2, which was age‐upregulated in all five datasets from the brain, as well as a handful of datasets from heart, muscle, and immune tissues. Collectively, these findings illustrate how the meta‐analysis ranking system was able to reveal genes with striking age‐related expression patterns.

### 3.3 Gene Ontology Patterns Were Consistent With Previous Literature

**FIGURE 2:**
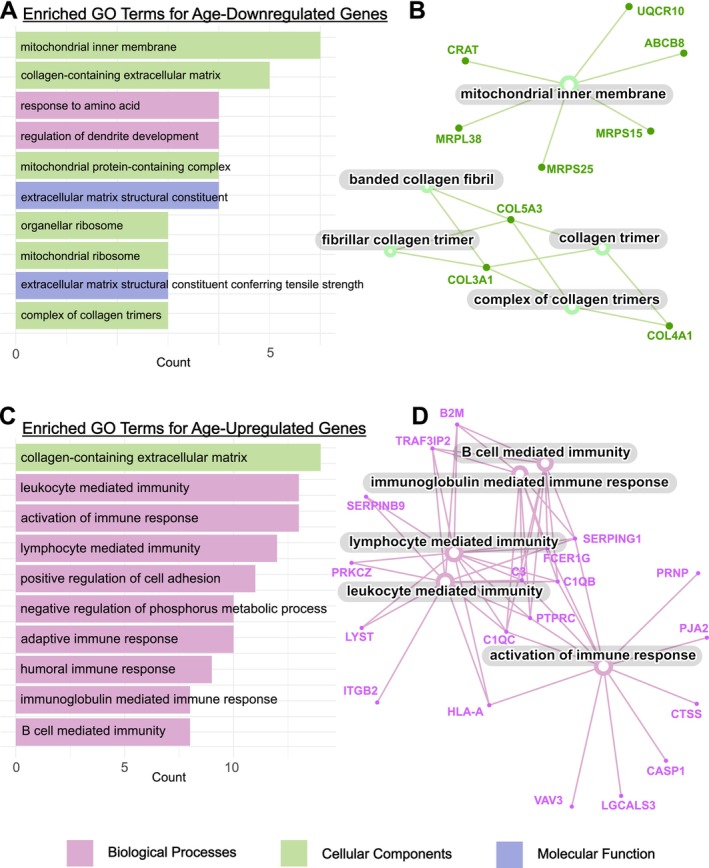
Gene ontology (GO) enrichment analysis of the 130 genes ranked highly for consistent differential expression with age, including 40 age‐downregulated genes and 90 age‐upregulated genes. For each group, bar charts display the top ten GO terms, colored according to three major GO term categories shown in the key. (A) Among age‐downregulated genes, the most well‐represented GO category was Cellular Components (CC, green). (B) The CC category is displayed in more detail in the accompanying network diagram, highlighting the downregulation of collagen‐related and mitochondrial membrane‐related genes. (C) Among age‐upregulated genes, the major category was Biological Processes (BP, red). (D) The BP category is expanded to show the variety of immune response‐related genes upregulated with age. The data were analyzed and visualized in R using the *clusterProfiler* package standard settings including Benjamini‐Hochberg adjusted *p* < 0.05.

Gene ontology (GO) enrichment analysis was performed to assess how high‐ranking DEGs could be categorized into recognizable functional groups and pathways. For this analysis, the cutoff was relaxed to include DEGs of rank 6 and above, yielding a pool of 40 age‐downregulated and 90 age‐upregulated genes. The GO term matching the largest number of genes from the downregulated pool was the mitochondrial inner membrane, and several additional terms related to mitochondria were enriched as well (Figure 2A,B). Also strongly represented were both cellular components and molecular functions related to extracellular matrix (ECM) proteins, particularly collagens. Of note, collagen‐containing extracellular matrix is one of the largest, broadest GO terms, with nearly 400 member genes, and included both age‐downregulated and age‐upregulated genes in our analysis. Notable age‐upregulated genes in this group were enzymes involved in cleaving and cross‐linking ECM components, such as transglutaminase 2. The overwhelming majority of the GO terms enriched among age‐upregulated genes were biological processes related to immune activity, especially adaptive immunity (Figure 2C,D). The results of this pathway analysis largely aligned with expectations and patterns observed in previous studies, reinforcing the validity of the meta‐analysis design and execution.

### 3.4 Knocking Down Orthologs of Several Mammalian DEGs Extended Lifespan in *C. elegans*

To prepare for these in vivo experiments, *C. elegans* orthologs of the highest‐ranking mammalian DEGs were identified using OrthoList 2, and corresponding RNAi clones were cultured and verified by Sanger sequencing. Of the 16 age‐downregulated DEGs, 11 (69%) were conserved in *C. elegans* , and verified RNAi clones for 9 of these orthologs were successfully cultured (Table S2). Of the 29 age‐upregulated DEGs, 16 (55%) were conserved, and 10 clones were obtained (Table S3). In total, 19 RNAi clones were prepared for knock‐down experiments.

To examine the effect of each gene on organismal senescence regardless of any role in embryonic and juvenile development, bacteria carrying each of these 19 RNAi clones were fed to the worms post‐developmentally, and the impact on lifespan was recorded. Post‐developmental knockdown of five of the nine age‐downregulated genes significantly extended lifespan relative to negative controls during the initial screening experiments (≥ 5% lifespan extension, *p* < 0.05 by log‐rank test, *n* = 80–100, Table S2). Knockdown of *C42C1.8*, ortholog of DIRC2, produced the largest effect at 50% extension. Of the ten age‐upregulated genes tested, four significantly extended lifespan, with EFEMP1 exhibiting the largest effect at 45% extension (Table S3). All experiments yielding statistically significant results were later repeated in an independent validation experiment using a single freshly thawed colony of worms to mitigate genetic drift and inter‐batch variability (*n* = 100–150).

**FIGURE 3:**
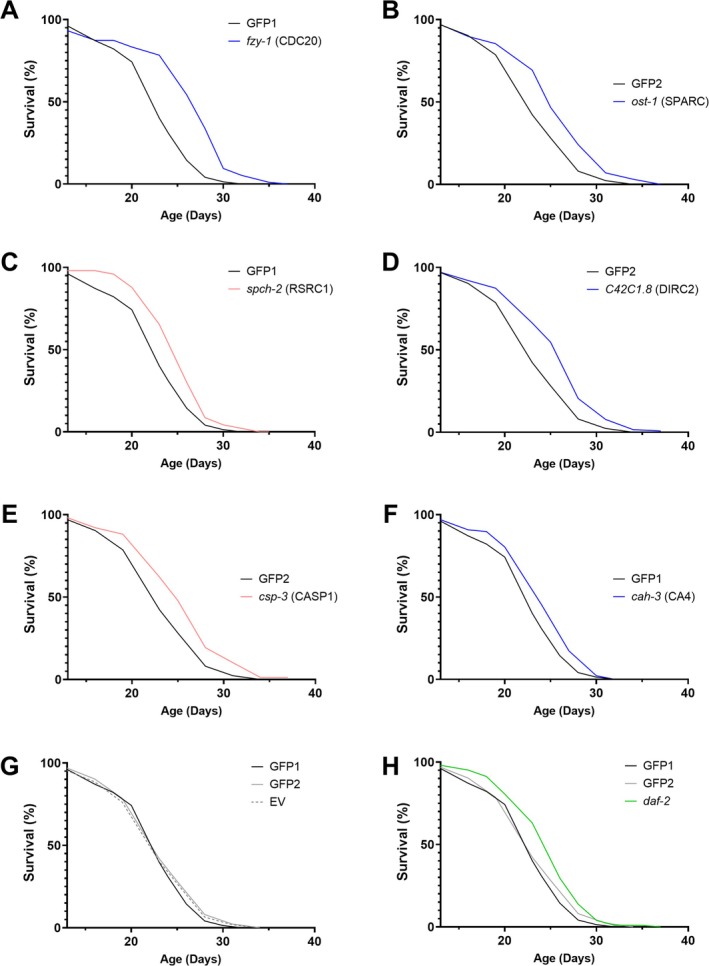
*Caenorhabditis elegans* survival curves for lifespan‐extending post‐developmental RNAi targeting orthologs of mammalian DEGs, including age‐downregulated (blue) and age‐upregulated (red) genes. Each RNAi clone significantly extended lifespan (A–F, log‐rank test, Bonferroni‐adjusted *p* < 0.05) in two independent experiments, and the results of the second experiment are shown here (*n* ≈ 100–150 worms per group). This experiment was performed in two back‐to‐back batches with an internal GFP control group in each batch (GFP1 for batch 1, GFP2 for batch 2). (G) Negative control GFP groups performed very similarly to each other (solid lines) and to the alternative empty L4440 vector control (dotted lines). (H) The RNAi clone Ahringer III‐7G14 against *daf‐2* was used a positive control (green). For detailed quantification, see Table 2.

A total of six RNAi clones significantly extended lifespan in the validation experiments as well as in the initial screenings. In order from largest to smallest mean lifespan extension, these RNAi knockdowns targeted: *fzy‐1* (ortholog of CDC20), *ost‐1* (SPARC), *spch‐2* (RSRC1), *C42C1.8* (DIRC2/SLC49A4), *csp‐3* (CASP1), and *cah‐3* (CA4) (Figure 3A–F). The two age‐upregulated targets were *spch‐2* (RSRC1) and *csp‐3* (CASP1), whereas the other four targets were age‐downregulated. Mean lifespan extension ranged from 9% to 15%, median extension from 6% to 19%, and maximum lifespan from 4% to 15% relative to within‐batch GFP controls, which averaged a lifespan mean of 23.35, median 22.14, and maximum 27.29 days (Table 2). The validation experiments demonstrated minimal variability, evidenced by the overlapping survival curves of the three independent negative control groups (Figure 3G). The degree of lifespan extension was comparable to the positive control RNAi against *daf‐2* (Figure 3H), which extended mean lifespan by 9%, median by 9%, and maximum by 7% (*n* = 103, log‐rank test, Bonferroni‐adjusted *p* < 0.01, Table 2).

**TABLE 2:** Lifespan extension in *Caenorhabditis elegans* via post‐developmental RNAi of orthologs of mammalian DEGs.

Human DEG	Worm Ortholog (Clone)	Number subjects	Mean lifespan ± Standard error (%Extension)	Median lifespan (%Extension)	Maximum lifespan (%Extension)	Corrected *p* value
CDC20 ↓	*fzy‐1* (II‐4O16)	103	26.21 ± 0.56 (15%)	26.41 (19%)	29.96 (12%)	< 0.001
SPARC ↓	*ost‐1* (IV‐9H03)	143	26.38 ± 0.46 (11%)	24.91 (12%)	31.89 (15%)	< 0.001
DIRC2 ↓	*C42C1.8* (IV‐6D23)	151	26.09 ± 0.4 (10%)	25.41 (15%)	30.47 (10%)	< 0.001
CA4 ↓	*cah‐3* (X‐7 M16)	98	24.66 ± 0.44 (8%)	23.48 (6%)	28.45 (6%)	< 0.001
RSRC1 ↑	*spch‐2* (I‐3C03)	99	25.31 ± 0.38 (11%)	24.31 (10%)	27.87 (4%)	< 0.001
CASP1 ↑	*csp‐3* (I‐5P02)	101	25.93 ± 0.49 (9%)	24.7 (12%)	31.06 (12%)	< 0.01
Positive Control	*daf‐2* (III‐7G14)	103	24.95 ± 0.42 (9%)	24.17 (9%)	28.77 (7%)	< 0.01

*Note:* DEGs are written with HUGO (Human Genome Organization) Gene Nomenclature Committee (HGNC) symbols and an arrow indicating whether the gene was age‐downregulated or age‐upregulated. Worm orthologs are written using the common name if available, otherwise the sequence name, followed in parentheses by the specific Ahringer RNAi clone utilized. Percent lifespan extension was calculated against internal GFP controls using the log‐rank test with Bonferroni adjusted *p* values. Average GFP control lifespan was mean = 23.35, median = 22.14, maximum = 27.29 days.

Abbreviation: DEG, differentially expressed gene.

### 3.5 Knocking Down Orthologs of Several Mammalian DEGs Extended Lifespan in *C. elegans*

Lastly, the expression patterns of the six genes that consistently extended lifespan in worms were re‐examined in our mammalian datasets (Figure S2). CASP1 and RSCR1 were age‐upregulated in seven datasets each with no age‐downregulation. CA4, DIRC2, and CDC20 were age‐downregulated in seven or eight datasets each with no age‐upregulation. SPARC was age‐downregulated in nine datasets but also age‐upregulated in one dataset derived from mouse liver. All six were differentially expressed with age in multiple mouse tissues; all except CDC20 were also differentially expressed in human tissues; and all except CA4 also in rat tissues. All six genes were differentially expressed in at least one of the seven datasets from muscle, which is unsurprising, but also one of only two datasets from liver, which is a much higher rate. Expression patterns in adipose tissue were also prominent: CASP1, RSCR1, and SPARC were differentially expressed in two of three fat datasets, and CA4 and DIRC2 in one dataset each. Finally, RSCR1, CA4, and CDC20 were differentially expressed in certain brain tissues including human frontal cortex and mouse neocortex and striatum. In summary, the most striking pattern was that knockdown of age‐upregulated DEGs was not more likely than age‐downregulated DEGs to extend lifespan, and the prominent contributions from adipose and liver tissue were also notable.

## 4 Discussion

This study has established a geroscience‐specific workflow to channel large quantities of gene expression data into a streamlined list of actionable targets using accessible, scalable tools in computational biology and *C. elegans* research. The goal of this approach is to maximize the value of existing research by harnessing these readily available datasets and methods effectively to produce novel, valuable discoveries.

Over the past few decades, there have been copious studies comparing gene expression in tissues from older versus younger subjects in a variety of species (Zahn et al. 2007; de Magalhães et al. 2024), and these generally culminate in conclusions based on functional enrichment analysis. In general, advanced age has been associated with upregulation of immune and inflammatory pathways but downregulation of the electron transport chain and other mitochondrial activities as well as collagens and other structural ECM proteins (de Magalhães et al. 2009; Zahn et al. 2007; Peters et al. 2015), and our results were consistent with these established trends. However, therapeutic directions cannot be extrapolated from purely observational gene expression data, where drivers of aging cannot be distinguished from compensatory protective responses and irrelevant downstream effects. Moreover, there is no guarantee that functional groups reflect concerted biological activities; for example, the GO term collagen‐containing ECM is broad enough to encompass both age‐downregulated structural collagens, which are synthesized less effectively by aging cells, as well as age‐upregulated proteases and cross‐linking proteins such as transglutaminases, which classically drive fibrosis and stiffening in aging tissues (Park et al. 2023). Finally, functional enrichment analyses are biased in favor of well‐defined gene sets and by definition exclude novel, undiscovered functions from the results. It is important to consider these limitations and take a closer look at individual genes.

Two of our highest‐ranking individual genes, EFEMP1 (Rank 11) and CP (Rank 9), which were consistently age‐upregulated over all six major tissue types, have known associations with age‐related pathologies and have also been classified as age‐associated in previous similar meta‐analyses (de Magalhães et al. 2009; Palmer et al. 2021). EFEMP1, also known as fibulin‐3, is an ECM glycoprotein strongly associated with aging pathologies: overexpression contributes to age‐related macular degeneration, high plasma levels are associated with signs of brain aging and higher risk of dementia, and upregulation of this gene is associated with Werner syndrome, a premature aging condition (Cheng et al. 2020; McGrath et al. 2022). On the other hand, CP, or ceruloplasmin, is a copper‐binding glycoprotein involved in iron metabolism and defense against oxidative stress; decreased CP activity is associated with advanced age and age‐related diseases, such as Parkinson's and Alzheimer's disease (Semsei et al. 1993; Kristinsson et al. 2012; Wang and Wang 2019). From this context, we may infer that although both genes exhibit similar expression profiles, EFEMP1 likely plays a role driving age‐related pathology, whereas CP may be upregulated with age as a compensatory response to amplify its protective effects. However, even for well‐documented genes like these, such inferences still involve speculation, and there are many other DEGs that are much less clearly characterized without supplemental information.

We thus focused on funneling our DEGs into a *C. elegans* RNAi lifespan screen to gain insights on the role of each gene in aging and longevity. Two of the 10 tested age‐upregulated genes extended lifespan when knocked down in *C. elegans* : *csp‐3*, an ortholog of CASP1, and *spch‐2*, an ortholog of RSRC1.

Caspases are proteases involved in apoptosis and inflammation (Molla et al. 2020), and CASP‐1 is particularly well known as a major component of the NLRP3‐CASP1 inflammasome and a promising therapeutic target for Hutchinson‐Gilford progeria, another premature aging syndrome, and Alzheimer's disease (Heneka et al. 2013). Interestingly, CASP1 was not differentially expressed in any of the brain datasets we examined, which included samples from the human frontal cortex. However, there is evidence that CASP1 is overexpressed in the frontal cortex and hippocampus of patients with Alzheimer's disease (Heneka et al. 2013). The novel discovery that RNAi inhibition of an orthologous caspase extends lifespan in worms may suggest an evolutionarily conserved role for caspases in driving age‐related neurodegeneration beyond the canonical CASP1‐NLRP3 inflammasome, which is vertebrate‐specific. Supporting this hypothesis are studies in *C. elegans* demonstrating that suppressing caspase activity reduces age‐associated decline in neuronal signaling (Wirak et al. 2022). This may help explain the recent unexpected finding that pharmacological CASP‐1 inhibitors protect against cognitive decline in mouse models of Alzheimer dementia by altering neuronal function rather than modulating inflammation (Flores et al. 2020; Flores et al. 2022).

RSRC1, named for its structure, arginine and serine rich coiled‐coil 1, is a member of an evolutionarily conserved family of regulators of pre‐mRNA splicing. Recent advances in genetics have revealed RSRC1 mutations are associated with aberrant human brain development: RSRC1 polymorphism is associated with schizophrenia (Potkin et al. 2009), and patients homozygous for loss‐of‐function RSRC1 mutations exhibit developmental delay and intellectual disability (Perez et al. 2018; Scala et al. 2020). In our meta‐analysis, RSRC1 was age‐upregulated in three of the five datasets from brain tissue, and post‐developmental knockdown produced lifespan extension. These data suggest RSRC1 is involved in early brain development but functions aberrantly late in life as a driver of aging. This dichotomy is known as antagonistic pleiotropy, wherein natural selection favors alleles that confer an advantage early in life when selective pressure is strongest, even though they may produce deleterious effects late in life when selection is weakest (Austad and Kirkwood 2008; Williams 1957). The important role of alternative pre‐mRNA splicing in aging and longevity is well established and reviewed in detail elsewhere (Bhadra et al. 2020).

Four of the nine tested age‐downregulated genes we tested extended lifespan when knocked down in *C. elegans* , including orthologs of two of the four highest‐ranking (Rank 8) age‐downregulated DEGs: *ost‐1*, ortholog of SPARC; and *cah‐3*, an ortholog of CA4. Counteracting aging by further suppressing genes that were naturally downregulated with age is not intuitive. Instinct tells us that we must reverse age‐related changes in gene expression to preserve a healthy, youthful state. However, such changes are not necessarily deleterious. The classic example of a beneficial change is the rise in stress response genes such as APOD, further overexpression of which actually helps animals resist neurodegeneration and extend lifespan (Rassart et al. 2020; Muffat et al. 2008). There is less existing data on further suppressing age‐downregulated genes, but one example is the mitochondrial electron transport chain. Complex I components in particular decline with age across multiple species (de Magalhães et al. 2009; Zahn et al. 2007), yet further suppression of these components via RNAi extends lifespan in both flies (Copeland et al. 2009) and worms (Rea et al. 2007).

A possible explanation for this pattern is that natural downregulation of these genes may be seen as an adaptive response to aging, and further suppression is simply boosting this natural adaptation, a concept similar to the aforementioned APOD example. There is also the concept of hormesis, wherein provoking a minor stress can trigger a major compensatory response that nets a favorable result. For example, RNAi perturbations of the electron transport chain stimulate the expression of cell‐protective genes via a process called retrograde response (mitohormesis) (Ristow and Zarse 2010; Cristina et al. 2009). This underscores the importance of considering the function of each gene individually rather than assuming its role based on expression pattern alone.

The first age‐downregulated gene for our consideration is SPARC (Secreted protein acidic and rich in cysteine). Also known as osteonectin, SPARC is a highly conserved ECM glycoprotein that regulates collagen maturation and cell‐matrix interactions (Fitzgerald and Schwarzbauer 1998; Toba and Takai 2024). SPARC is expressed ubiquitously in mammalian tissues, particularly in adipocytes, and is involved in bone development and turnover and wound healing, especially in corneal tissue; SPARC KO mice have dysregulated collagen and suffer from osteopenia and cataracts (Rosset and Bradshaw 2016; Basu et al. 2001; Lin et al. 2023; Kos and Wilding 2010). However, recent studies have revealed pathologic roles for SPARC in adulthood, such as adipose fibrosis, age‐related inflammation, metabolic dysfunction, obesity, and diabetes and diabetic nephropathy and retinopathy (Kos and Wilding 2010; Xu et al. 2013; Ryu et al. 2022). Studies in worms too have shown that although SPARC is crucial for development (Fitzgerald and Schwarzbauer 1998), overexpression disrupts extracellular collagen trafficking and reduces incorporation of collagens into the basement membrane (Toba and Takai 2024) and that collagen dynamics are a key regulator of longevity (Teuscher et al. 2024). In our meta‐analysis, SPARC was downregulated with age in all major tissues studied except the brain and liver; the most pronounced pattern was in adipose tissue, where expression significantly decreased with age in two of the three datasets. We speculate that SPARC expression may be reduced with age when there is less need to mature new collagen fibrils and more risk of contributing to age‐related tissue fibrosis. Our finding that SPARC knockdown extends lifespan suggests that SPARC plays a predominantly detrimental role in the post‐developmental period by contributing to the stiffening of the ECM in both invertebrates and higher organisms, and thus augmenting the natural decline in SPARC expression is beneficial.

The next age‐downregulated gene is carbonic anhydrase 4 (CA4). Carbonic anhydrases are crucial for regulating pH, a fundamental biological process, and they are ubiquitous from microbes to mammals (Aspatwar et al. 2022). Carbonic anhydrase inhibitors, such as acetazolamide, have been pursued as potential treatments for many conditions including the age‐related diseases glaucoma, Alzheimer's dementia, and cancer (Aspatwar et al. 2022; Solesio et al. 2018). We found one member of this enzyme family, CA4, to be consistently downregulated with age across all six major tissue types studied, including human and mouse brain datasets. CA4 is predominantly expressed in the brain, colon, and lung (Aspatwar et al. 2022). This particular carbonic anhydrase has been studied for its role in extracellular buffering in the central nervous system, especially the hippocampus and retina, and mutations are known to cause retinitis pigmentosa in humans (Yang et al. 2005; Ghandour et al. 1992; Shah et al. 2005). In adulthood, however, increased CA4 expression has recently been linked to dystrophic calcification of the ECM resulting in stiffening of airway cartilage in chronic obstructive pulmonary disease (COPD) (Nava et al. 2022). The premise that carbonic anhydrase inhibition could reverse age‐related calcification was explored by administering acetazolamide to klotho‐hypomorphic mice, a model of accelerated aging, and treatment successfully ameliorated calcification and tripled lifespan (Leibrock et al. 2016). Consistent with this premise, we showed for the first time that post‐developmentally downregulating the CA4 ortholog, *cah‐3*, extended lifespan in *C. elegans* . In sum, CA4 is important for regulating pH during central nervous development but may contribute to pathological tissue stiffening in adulthood, especially in the lungs.

The largest lifespan extension achieved in our study was via RNAi knockdown of *fzy‐1*, ortholog of CDC20, which was age‐downregulated in our meta‐analysis. Cell division cycle 20 (CDC20) is an evolutionarily conserved, positive regulator of cell division essential for life in both worms and mammals (Kamath et al. 2003; Li et al. 2007). The activity of CDC20 must be tightly regulated, as hyperactivity is associated with aneuploidy, leading to premature aging and oncogenesis (Clarke et al. 2003; Fujita et al. 2020; Wang et al. 2015), whereas suppression has recently been linked to cellular senescence (Volonte et al. 2022). Out of the six lifespan‐extending RNAi interventions we identified in this study, *fzy‐1* is the only one that was not novel, as this was previously demonstrated by Xue et al. (2007) in their study on network models of aging. The mechanism for *fzy‐1* remains unknown, but post‐developmental inhibition of other cell cycle factors in *C. elegans* is thought to produce lifespan extension via well‐known longevity pathways (Dottermusch et al. 2016), namely the metabolic pathway via *daf‐16* (ortholog of FOXO) (Zečić and Braeckman 2020) and the stress response pathway via *skn‐1* (ortholog of NRF) (Blackwell et al. 2015). As *C. elegans* is a post‐mitotic organism, it is interesting to contemplate the implications of aberrant reentry into the cell cycle: in humans, neuronal cell cycle reentry is thought to be critical for development but contributes to brain aging and neurodegeneration in adulthood (Wu et al. 2024; Becker and Bonni 2004), and it is plausible that *fzy‐1* RNAi could rescue *C. elegans* from a similar phenomenon. That said, these findings should be interpreted with caution, as it is also plausible that CDC20 downregulation could be maladaptive in humans by promoting cellular senescence.

Lastly, we found that post‐developmental knockdown of the DIRC2 ortholog *C42C1.8* extends lifespan in *C. elegans* . Disrupted in renal carcinoma 2 (DIRC2), also known as solute carrier family 49 member 4 (SLC49A4), is an evolutionarily conserved putative transporter enriched in lysosomal membranes (Bodmer et al. 2002). Aside from its eponymous involvement in renal carcinogenesis, little was known about DIRC2 until 2023, when Akino et al. (2023) demonstrated that it is an H^+^‐driven lysosomal pyridoxine (vitamin B6) exporter. Downregulation of DIRC2 is expected to impair this lysosomal function, reducing the cytosolic availability of vitamin B6. This year, a GWAS study of long‐lived dogs found that DIRC2 was one of the nine genes associated with longevity (Korec et al. 2025). Our work demonstrates that DIRC2 is actually one of the most consistently age‐downregulated genes in mammals, with expression declining in both human and mouse muscle tissues and rodent fat, heart, liver, and trachea. Given that lysosomal activity and vitamin B6 availability are both generally considered pro‐longevity (Kato et al. 2024; Tan and Finkel 2023), we speculate that a decline in DIRC2 that disrupts these factors may drive age‐related dysfunction. In that case, our RNAi results could be explained by a hormesis phenomenon related to mitohormesis, wherein minor mitochondrial stresses promote longevity (Ristow and Zarse 2010); indeed, there is evidence that lysosomal signaling promotes longevity via adjustments in mitochondrial activity (Ramachandran et al. 2019). However, this is speculative at this stage. The example of DIRC2 epitomizes the power of our combined DEG meta‐analysis and functional *C. elegans* screen to identify promising, understudied targets for further investigation.

*Caenorhabditis elegans* has long been used to investigate the mechanisms of aging using well‐developed functional genomics tools. There were two genome‐wide RNAi longevity screens, by the Ruvkun (Hamilton et al. 2005) and Kenyon (Hansen et al. 2005) groups, each boasting 70%–80% coverage of all open reading frames. Due to very high false negative rates, they identified a combined total of 120 longevity genes, with only four genes in common (Petrascheck and Miller 2017). The Ruvkun group performed a follow‐up screen using post‐developmental instead of embryonic RNAi on 2700 genes essential for development (Curran and Ruvkun 2007), and similar smaller studies have been published by others since (Chen et al. 2007; Tacutu et al. 2012). The yield of lifespan‐extending gene activations out of total genes tested was < 1% for genome‐wide screens and 2.4% for the post‐developmental screen of essential genes, reflecting the importance of antagonistic pleiotropy in aging (Austad and Kirkwood 2008; Williams 1957). A more recent study achieved a yield of 44% when testing orthologs of genes differentially expressed with age in human blood, and this study also reported a background rate of 7% yield for randomly chosen genes (Sutphin et al. 2017). Yield is highly dependent on experimental methods such as the number of animals and time points as well as environmental factors like temperature; in this latter case, the authors also tested several of the genes under two different conditions (pre‐ and post‐developmentally), raising the yield. Here we reported a yield of 32%, suggesting that roughly one third of the candidates identified in our meta‐analysis are drivers of aging; the remaining two thirds may have negligible or protective effects, or they may also be drivers of aging but under conditions not tested in this experiment.

There are important limitations to our study, many of which pertain to the nature of RNAi screens and the challenges of modeling human physiology in worms. First, as there is not an equivalently resource‐light method for overexpression screens, we rely on RNAi knockdown only, and thus our study cannot detect positive effects of genes on longevity. Secondly, only some of the high‐ranking DEGs corresponded to verifiable worm orthologs and were able to be tested, and even those orthologs were selected with varying levels of confidence and specificity. This means that failure of a candidate gene to extend lifespan in our study does not indicate that the candidate is a poor subject for further research. Finally, the evolutionary distance between humans and worms warrants caution in our interpretations of the functional roles of each gene product, as molecules with similar structures can play different biological roles in such distinct species.

It should also be noted that the datasets included in our meta‐analysis were derived from neither a complete nor an even distribution of tissue types; for example, there were no datasets derived from the kidneys or intestines, whereas muscle was highly represented. As more datasets are made available, we expect this approach to provide increasing contributions to the geroscience literature. Consistent with this goal, the methods described herein are intended to be accessible and flexible enough for others to reproduce and expand our workflow in future studies. Only basic coding skills in R and Python are required to reproduce the meta‐analysis, and the GEO2R toolset at the core of our scripts has recently been updated to accept both microarray and RNAseq datasets.

In conclusion, the overall trends we observed in our meta‐analysis were consistent with previous literature, but our novel workflow identified six genes with evolutionarily conserved causal roles in the aging process. Of note, knocking down age‐upregulated genes was not more likely to produce life extension than interfering with age‐downregulated genes. Thus, our results do not support the commonly held assumption that reversing any changes in age‐related gene expression is beneficial, and future studies should further investigate this trend.

## Author Contributions

Conceptualization and methodology: Ariella Coler‐Reilly, Zachary Pincus; Formal analysis and investigation: Ariella Coler‐Reilly, Erica L. Scheller, Roberto Civitelli; Writing – original draft preparation: Ariella Coler‐Reilly; Writing – review and editing: Zachary Pincus, Erica L. Scheller, Roberto Civitelli; Funding acquisition: Ariella Coler‐Reilly, Roberto Civitelli; Resources: Zachary Pincus, Roberto Civitelli; Supervision: Erica L. Scheller, Roberto Civitelli.

## Conflicts of Interest

The authors declare no conflicts of interest.

## Supporting information


**Table S1:** Datasets used in the meta‐analysis of genes differentially expressed during mammalian aging.
**Table S2:** Experimental outcomes of the most consistently age‐downregulated mammalian genes in *C. elegans* .
**Table S3:** Experimental outcomes of the most consistently age‐upregulated mammalian genes in *C. elegans* .
**Figure S1:** The number of datasets and DEGs derived from each species and tissue type.
**Figure S2:** Bubble plots showing the expression patterns of the six drivers of aging identified in this study: CASP1, RSRC1, SPARC, CA4, CDC20, and DIRC2.

## Data Availability

The gene expression datasets analyzed in this study were obtained from publicly available sources, specifically the National Center for Biotechnology Information (NCBI) Gene Expression Omnibus (GEO). A full list of datasets, including accession numbers, is provided in Table S1. The scripts used for meta‐analysis and data processing are permanently stored and available at GitHub Repository: AriellaStudies/Aging‐DEGs.

## References

[acel70225-bib-0001] Akino, S. , T. Yasujima , T. Yamashiro , and H. Yuasa . 2023. “Disrupted in Renal Carcinoma 2 (DIRC2/SLC49A4) is an H+‐Driven Lysosomal Pyridoxine Exporter.” Life Science Alliance 6, no. 2: e202201629. 10.26508/lsa.202201629. 36456177 PMC9719028

[acel70225-bib-0002] Aspatwar, A. , M. E. E. Tolvanen , H. Barker , et al. 2022. “Carbonic Anhydrases in Metazoan Model Organisms: Molecules, Mechanisms, and Physiology.” Physiological Reviews 102, no. 3: 1327–1383. 10.1152/physrev.00018.2021. 35166161

[acel70225-bib-0003] Austad, S. N. , and T. Kirkwood . 2008. “Evolutionary Theory in Aging Research.” In Cold Spring Harbor Monograph. Molecular Biology of Aging, edited by L. Guarente , L. Partridge , and D. C. Wallace , vol. 51, 95–111. Cold Spring Harbor Laboratory Press.

[acel70225-bib-0004] Bahar, R. , C. H. Hartmann , K. A. Rodriguez , et al. 2006. “Increased Cell‐To‐Cell Variation in Gene Expression in Ageing Mouse Heart.” Nature 441, no. 7096: 1011–1014. 10.1038/nature04844. 16791200

[acel70225-bib-0005] Barrett, T. , S. E. Wilhite , P. Ledoux , et al. 2013. “NCBI GEO: Archive for Functional Genomics Data Sets–Update.” Nucleic Acids Research 41, no. Database issue: D991–D995. 10.1093/nar/gks1193. 23193258 PMC3531084

[acel70225-bib-0006] Basu, A. , L. H. Kligman , S. J. Samulewicz , and C. C. Howe . 2001. “Impaired Wound Healing in Mice Deficient in a Matricellular Protein SPARC (Osteonectin, BM‐40).” BMC Cell Biology 2, no. 1: 15. 10.1186/1471-2121-2-15. 11532190 PMC48139

[acel70225-bib-0007] Becker, E. B. E. , and A. Bonni . 2004. “Cell Cycle Regulation of Neuronal Apoptosis in Development and Disease.” Progress in Neurobiology 72, no. 1: 1–25. 10.1016/j.pneurobio.2003.12.005. 15019174

[acel70225-bib-0008] Bhadra, M. , P. Howell , S. Dutta , C. Heintz , and W. B. Mair . 2020. “Alternative Splicing in Aging and Longevity.” Human Genetics 139, no. 3: 357–369. 10.1007/s00439-019-02094-6. 31834493 PMC8176884

[acel70225-bib-0009] Blackwell, T. K. , M. J. Steinbaugh , J. M. Hourihan , C. Y. Ewald , and M. Isik . 2015. “SKN‐1/Nrf, Stress Responses, and Aging in *Caenorhabditis elegans* .” Free Radical Biology & Medicine 88, no. Pt B: 290–301. 10.1016/j.freeradbiomed.2015.06.008. 26232625 PMC4809198

[acel70225-bib-0010] Bodmer, D. , M. Eleveld , E. Kater‐Baats , et al. 2002. “Disruption of a Novel MFS Transporter Gene, DIRC2, by a Familial Renal Cell Carcinoma‐Associated t(2;3)(q35;q21).” Human Molecular Genetics 11, no. 6: 641–649. 10.1093/hmg/11.6.641. 11912179

[acel70225-bib-0011] Chen, C.‐H. , Y.‐C. Chen , H.‐C. Jiang , C.‐K. Chen , and C.‐L. Pan . 2013. “Neuronal Aging: Learning From *C. elegans* .” Journal of Molecular Signaling 8, no. 1: 14. 10.1186/1750-2187-8-14. 24325838 PMC3895751

[acel70225-bib-0012] Chen, D. , K. Z. Pan , J. E. Palter , and P. Kapahi . 2007. “Longevity Determined by Developmental Arrest Genes in *Caenorhabditis elegans* .” Aging Cell 6, no. 4: 525–533. 10.1111/j.1474-9726.2007.00305.x. 17521386 PMC2746107

[acel70225-bib-0013] Cheng, L. , C. Chen , W. Guo , et al. 2020. “EFEMP1 Overexpression Contributes to Neovascularization in Age‐Related Macular Degeneration.” Frontiers in Pharmacology 11: 547436. 10.3389/fphar.2020.547436. 33584252 PMC7874111

[acel70225-bib-0014] Clarke, D. J. , M. Segal , C. A. Andrews , et al. 2003. “S‐Phase Checkpoint Controls Mitosis via an APC‐Independent Cdc20p Function.” Nature Cell Biology 5, no. 10: 928–935. 10.1038/ncb1046. 14502293

[acel70225-bib-0015] Copeland, J. M. , J. Cho , T. Lo Jr. , et al. 2009. “Extension of Drosophila Life Span by RNAi of the Mitochondrial Respiratory Chain.” Current Biology: CB 19, no. 19: 1591–1598. 10.1016/j.cub.2009.08.016. 19747824

[acel70225-bib-0016] Cristina, D. , M. Cary , A. Lunceford , C. Clarke , and C. Kenyon . 2009. “A Regulated Response to Impaired Respiration Slows Behavioral Rates and Increases Lifespan in *Caenorhabditis elegans* .” PLoS Genetics 5, no. 4: e1000450. 10.1371/journal.pgen.1000450. 19360127 PMC2660839

[acel70225-bib-0017] Curran, S. P. , and G. Ruvkun . 2007. “Lifespan Regulation by Evolutionarily Conserved Genes Essential for Viability.” PLoS Genetics 3, no. 4: e56. 10.1371/journal.pgen.0030056. 17411345 PMC1847696

[acel70225-bib-0018] Davis, S. , and P. S. Meltzer . 2007. “GEOquery: A Bridge Between the Gene Expression Omnibus (GEO) and BioConductor.” Bioinformatics (Oxford, England) 23, no. 14: 1846–1847. 10.1093/bioinformatics/btm254. 17496320

[acel70225-bib-0019] de Magalhães, J. P. , Z. Abidi , G. A. dos Santos , et al. 2024. “Human Ageing Genomic Resources: Updates on Key Databases in Ageing Research.” Nucleic Acids Research 52, no. D1: D900–D908. 10.1093/nar/gkad927. 37933854 PMC10767973

[acel70225-bib-0020] de Magalhães, J. P. , J. Curado , and G. M. Church . 2009. “Meta‐Analysis of Age‐Related Gene Expression Profiles Identifies Common Signatures of Aging.” Bioinformatics (Oxford, England) 25, no. 7: 875–881. 10.1093/bioinformatics/btp073. 19189975 PMC2732303

[acel70225-bib-0021] de Magalhães, J. P. , and O. Toussaint . 2004. “How Bioinformatics Can Help Reverse Engineer Human Aging.” Ageing Research Reviews 3, no. 2: 125–141. 10.1016/j.arr.2003.08.006. 15177050

[acel70225-bib-0022] Dillin, A. , D. K. Crawford , and C. Kenyon . 2002. “Timing Requirements for Insulin/IGF‐1 Signaling in *C. elegans* .” Science 298, no. 5594: 830–834. 10.1126/science.1074240. 12399591

[acel70225-bib-0023] Dottermusch, M. , T. Lakner , T. Peyman , M. Klein , G. Walz , and E. Neumann‐Haefelin . 2016. “Cell Cycle Controls Stress Response and Longevity in *C. elegans* .” Aging 8, no. 9: 2100–2126. 10.18632/aging.101052. 27668945 PMC5076454

[acel70225-bib-0024] Edgar, R. , M. Domrachev , and A. E. Lash . 2002. “Gene Expression Omnibus: NCBI Gene Expression and Hybridization Array Data Repository.” Nucleic Acids Research 30, no. 1: 207–210. 10.1093/nar/30.1.207. 11752295 PMC99122

[acel70225-bib-0025] Fitzgerald, M. C. , and J. E. Schwarzbauer . 1998. “Importance of the Basement Membrane Protein SPARC for Viability and Fertility in *Caenorhabditis elegans* .” Current Biology 8, no. 23: 1285–1288. 10.1016/s0960-9822(07)00540-4. 9822581

[acel70225-bib-0026] Flores, J. , M.‐L. Fillion , and A. C. LeBlanc . 2022. “Caspase‐1 Inhibition Improves Cognition Without Significantly Altering Amyloid and Inflammation in Aged Alzheimer Disease Mice.” Cell Death & Disease 13, no. 10: 864. 10.1038/s41419-022-05290-x. 36220815 PMC9553979

[acel70225-bib-0027] Flores, J. , A. Noël , B. Foveau , O. Beauchet , and A. C. LeBlanc . 2020. “Pre‐Symptomatic Caspase‐1 Inhibitor Delays Cognitive Decline in a Mouse Model of Alzheimer Disease and Aging.” Nature Communications 11, no. 1: 4571. 10.1038/s41467-020-18405-9. PMC7486940 32917871

[acel70225-bib-0028] Fujita, H. , T. Sasaki , T. Miyamoto , et al. 2020. “Premature Aging Syndrome Showing Random Chromosome Number Instabilities With CDC20 Mutation.” Aging Cell 19, no. 11: e13251. 10.1111/acel.13251. 33094908 PMC7681047

[acel70225-bib-0029] Ghandour, M. S. , O. K. Langley , X. L. Zhu , A. Waheed , and W. S. Sly . 1992. “Carbonic Anhydrase IV on Brain Capillary Endothelial Cells: A Marker Associated With the Blood‐Brain Barrier.” Proceedings of the National Academy of Sciences of the United States of America 89, no. 15: 6823–6827. 10.1073/pnas.89.15.6823. 1495971 PMC49596

[acel70225-bib-0030] Hamilton, B. , Y. Dong , M. Shindo , et al. 2005. “A Systematic RNAi Screen for Longevity Genes in *C. elegans* .” Genes & Development 19, no. 13: 1544–1555. 10.1101/gad.1308205. 15998808 PMC1172061

[acel70225-bib-0031] Han, S. K. , D. Lee , H. Lee , et al. 2016. “OASIS 2: Online Application for Survival Analysis 2 With Features for the Analysis of Maximal Lifespan and Healthspan in Aging Research.” Oncotarget 7, no. 35: 56147–56152. 10.18632/oncotarget.11269. 27528229 PMC5302902

[acel70225-bib-0032] Hansen, M. , A.‐L. Hsu , A. Dillin , and C. Kenyon . 2005. “New Genes Tied to Endocrine, Metabolic, and Dietary Regulation of Lifespan From a *Caenorhabditis elegans* Genomic RNAi Screen.” PLoS Genetics 1, no. 1: 119–128. 10.1371/journal.pgen.0010017. 16103914 PMC1183531

[acel70225-bib-0033] Heneka, M. T. , M. P. Kummer , A. Stutz , et al. 2013. “NLRP3 Is Activated in Alzheimer's Disease and Contributes to Pathology in APP/PS1 Mice.” Nature 493, no. 7434: 674–678. 10.1038/nature11729. 23254930 PMC3812809

[acel70225-bib-0034] Hou, L. , D. Wang , H. Cheng , B. Xian , and J.‐D. J. Han . 2016. “Systems Approaches to Understanding Aging.” In The Handbooks of Aging, Handbook of the Biology of Aging, edited by M. Kaeberlein and G. M. Martin , 241–261. Academic Press.

[acel70225-bib-0035] Huber, W. , V. J. Carey , R. Gentleman , et al. 2015. “Orchestrating High‐Throughput Genomic Analysis With Bioconductor.” Nature Methods 12, no. 2: 115–121. 10.1038/nmeth.3252. 25633503 PMC4509590

[acel70225-bib-0036] Hunter, J. D. 2007. “Matplotlib: A 2D Graphics Environment.” Computing in Science & Engineering 9, no. 3: 90–95. 10.1109/MCSE.2007.55.

[acel70225-bib-0037] Johnson, T. E. 2008. “ *Caenorhabditis elegans* 2007: The Premier Model for the Study of Aging.” Experimental Gerontology 43, no. 1: 1–4. 10.1016/j.exger.2007.09.008. 17977684 PMC2219387

[acel70225-bib-0038] Kamath, R. S. , and J. Ahringer . 2003. “Genome‐Wide RNAi Screening in *Caenorhabditis elegans* .” Methods 30, no. 4: 313–321. 10.1016/s1046-2023(03)00050-1. 12828945

[acel70225-bib-0039] Kamath, R. S. , A. G. Fraser , Y. Dong , et al. 2003. “Systematic Functional Analysis of the *Caenorhabditis elegans* Genome Using RNAi.” Nature 421, no. 6920: 231–237. 10.1038/nature01278. 12529635

[acel70225-bib-0040] Kato, N. , A. Kimoto , P. Zhang , et al. 2024. “Relationship of Low Vitamin B6 Status With Sarcopenia, Frailty, and Mortality: A Narrative Review.” Nutrients 16, no. 1: 177. 10.3390/nu16010177. 38202006 PMC10780671

[acel70225-bib-0041] Kennedy, B. K. , S. L. Berger , A. Brunet , et al. 2014. “Geroscience: Linking Aging to Chronic Disease.” Cell 159, no. 4: 709–713. 10.1016/j.cell.2014.10.039. 25417146 PMC4852871

[acel70225-bib-0042] Kim, W. , R. S. Underwood , I. Greenwald , and D. D. Shaye . 2018. “OrthoList 2: A New Comparative Genomic Analysis of Human and *Caenorhabditis elegans* Genes.” Genetics 210, no. 2: 445–461. 10.1534/genetics.118.301307. 30120140 PMC6216590

[acel70225-bib-0043] Kirkwood, T. B. , and S. N. Austad . 2000. “Why Do We Age?” Nature 408, no. 6809: 233–238. 10.1038/35041682. 11089980

[acel70225-bib-0044] Korec, E. , L. Ungrová , J. Kalvas , and J. Hejnar . 2025. “Identification of Genes Associated With Longevity in Dogs: 9 Candidate Genes Described in Cavalier King Charles Spaniel.” Veterinary and Animal Science 27: 100420. 10.1016/j.vas.2024.100420. 39823074 PMC11737349

[acel70225-bib-0045] Kos, K. , and J. P. H. Wilding . 2010. “SPARC: A Key Player in the Pathologies Associated With Obesity and Diabetes.” Nature Reviews. Endocrinology 6, no. 4: 225–235. 10.1038/nrendo.2010.18. 20195270

[acel70225-bib-0046] Kristinsson, J. , J. Snaedal , G. Tórsdóttir , and T. Jóhannesson . 2012. “Ceruloplasmin and Iron in Alzheimer's Disease and Parkinson's Disease: A Synopsis of Recent Studies.” Neuropsychiatric Disease and Treatment 8: 515–521. 10.2147/NDT.S34729. 23144563 PMC3493298

[acel70225-bib-0047] Leibrock, C. B. , I. Alesutan , J. Voelkl , et al. 2016. “Acetazolamide Sensitive Tissue Calcification and Aging of Klotho‐Hypomorphic Mice.” Journal of Molecular Medicine 94, no. 1: 95–106. 10.1007/s00109-015-1331-x. 26307633

[acel70225-bib-0048] Li, M. , J. P. York , and P. Zhang . 2007. “Loss of Cdc20 Causes a Securin‐Dependent Metaphase Arrest in Two‐Cell Mouse Embryos.” Molecular and Cellular Biology 27, no. 9: 3481–3488. 10.1128/MCB.02088-06. 17325031 PMC1899968

[acel70225-bib-0049] Lin, J. B. , X. Shen , C. W. Pfeifer , et al. 2023. “Dry Eye Disease in Mice Activates Adaptive Corneal Epithelial Regeneration Distinct From Constitutive Renewal in Homeostasis.” Proceedings of the National Academy of Sciences of the United States of America 120, no. 2: e2204134120. 10.1073/pnas.2204134120. 36595669 PMC9926235

[acel70225-bib-0050] Mancarci, O. n.d. “Homologene: Quick Access to Homologene and Gene Annotation Updates.” https://cran.r‐project.org/package=homologene.

[acel70225-bib-0051] McGrath, E. R. , J. J. Himali , D. Levy , et al. 2022. “Plasma EFEMP1 Is Associated With Brain Aging and Dementia: The Framingham Heart Study.” Journal of Alzheimer's Disease: JAD 85, no. 4: 1657–1666. 10.3233/JAD-215053. 34958018

[acel70225-bib-0052] McKinney, W. 2010. “Data Structures for Statistical Computing in Python.” In Proceedings of the 9th Python in Science Conference, Austin, Texas, 56–61. SciPy Proceedings. 10.25080/Majora-92bf1922-00a.

[acel70225-bib-0053] Molla, M. D. , Y. Akalu , Z. Geto , B. Dagnew , B. Ayelign , and T. Shibabaw . 2020. “Role of Caspase‐1 in the Pathogenesis of Inflammatory‐Associated Chronic Noncommunicable Diseases.” Journal of Inflammation Research 13: 749–764. 10.2147/JIR.S277457. 33116753 PMC7585796

[acel70225-bib-0054] Muffat, J. , D. W. Walker , and S. Benzer . 2008. “Human ApoD, an Apolipoprotein Up‐Regulated in Neurodegenerative Diseases, Extends Lifespan and Increases Stress Resistance in Drosophila.” Proceedings of the National Academy of Sciences of the United States of America 105, no. 19: 7088–7093. 10.1073/pnas.0800896105. 18458334 PMC2374552

[acel70225-bib-0055] Nava, V. E. , R. Khosla , S. Shin , F. E. Mordini , and B. C. Bandyopadhyay . 2022. “Enhanced Carbonic Anhydrase Expression With Calcification and Fibrosis in Bronchial Cartilage During COPD.” Acta Histochemica 124, no. 1: 151834. 10.1016/j.acthis.2021.151834. 34954529 PMC10312311

[acel70225-bib-0056] Palmer, D. , F. Fabris , A. Doherty , A. A. Freitas , and J. P. de Magalhães . 2021. “Ageing Transcriptome Meta‐Analysis Reveals Similarities and Differences Between Key Mammalian Tissues.” Aging 13, no. 3: 3313–3341. 10.18632/aging.202648. 33611312 PMC7906136

[acel70225-bib-0057] Park, J. Y. C. , A. King , V. Björk , B. W. English , A. Fedintsev , and C. Y. Ewald . 2023. “Strategic Outline of Interventions Targeting Extracellular Matrix for Promoting Healthy Longevity.” American Journal of Physiology. Cell Physiology 325, no. 1: C90–C128. 10.1152/ajpcell.00060.2023. 37154490

[acel70225-bib-0058] Perez, Y. , S. Menascu , I. Cohen , et al. 2018. “RSRC1 Mutation Affects Intellect and Behaviour Through Aberrant Splicing and Transcription, Downregulating IGFBP3.” Brain 141, no. 4: 961–970. 10.1093/brain/awy045. 29522154

[acel70225-bib-0059] Perez‐Gomez, A. , J. N. Buxbaum , and M. Petrascheck . 2020. “The Aging Transcriptome: Read Between the Lines.” Current Opinion in Neurobiology 63: 170–175. 10.1016/j.conb.2020.05.001. 32563038 PMC7484127

[acel70225-bib-0060] Peters, M. J. , R. Joehanes , L. C. Pilling , et al. 2015. “The Transcriptional Landscape of Age in Human Peripheral Blood.” Nature Communications 6: 8570. 10.1038/ncomms9570. PMC4639797 26490707

[acel70225-bib-0061] Petrascheck, M. , and D. L. Miller . 2017. “Computational Analysis of Lifespan Experiment Reproducibility.” Frontiers in Genetics 8: 92. 10.3389/fgene.2017.00092. 28713422 PMC5492194

[acel70225-bib-0062] Potkin, S. G. , J. A. Turner , J. A. Fallon , et al. 2009. “Gene Discovery Through Imaging Genetics: Identification of Two Novel Genes Associated With Schizophrenia.” Molecular Psychiatry 14, no. 4: 416–428. 10.1038/mp.2008.127. 19065146 PMC3254586

[acel70225-bib-0063] R Core Team . 2015. R: A Language and Environment for Statistical Computing. R Foundation for Statistical Computing. https://www.r‐project.org/.

[acel70225-bib-0064] Ramachandran, P. V. , M. Savini , A. K. Folick , et al. 2019. “Lysosomal Signaling Promotes Longevity by Adjusting Mitochondrial Activity.” Developmental Cell 48, no. 5: 685–696.e5. 10.1016/j.devcel.2018.12.022. 30713071 PMC6613828

[acel70225-bib-0065] Rangaraju, S. , G. M. Solis , R. C. Thompson , et al. 2015. “Suppression of Transcriptional Drift Extends *C. elegans* Lifespan by Postponing the Onset of Mortality.” eLife 4: e08833. 10.7554/eLife.08833. 26623667 PMC4720515

[acel70225-bib-0066] Rassart, E. , F. Desmarais , O. Najyb , K.‐F. Bergeron , and C. Mounier . 2020. “Apolipoprotein D.” Gene 756: 144874. 10.1016/j.gene.2020.144874. 32554047 PMC8011330

[acel70225-bib-0067] Rea, S. L. , N. Ventura , and T. E. Johnson . 2007. “Relationship Between Mitochondrial Electron Transport Chain Dysfunction, Development, and Life Extension in *Caenorhabditis elegans* .” PLoS Biology 5, no. 10: e259. 10.1371/journal.pbio.0050259. 17914900 PMC1994989

[acel70225-bib-0068] Rhodes, D. R. , J. Yu , K. Shanker , et al. 2004. “Large‐Scale Meta‐Analysis of Cancer Microarray Data Identifies Common Transcriptional Profiles of Neoplastic Transformation and Progression.” Proceedings of the National Academy of Sciences of the United States of America 101, no. 25: 9309–9314. 10.1073/pnas.0401994101. 15184677 PMC438973

[acel70225-bib-0069] Ristow, M. , and K. Zarse . 2010. “How Increased Oxidative Stress Promotes Longevity and Metabolic Health: The Concept of Mitochondrial Hormesis (Mitohormesis).” Experimental Gerontology 45, no. 6: 410–418. 10.1016/j.exger.2010.03.014. 20350594

[acel70225-bib-0070] Ritchie, M. E. , B. Phipson , D. Wu , et al. 2015. “Limma Powers Differential Expression Analyses for RNA‐Sequencing and Microarray Studies.” Nucleic Acids Research 43, no. 7: e47. 10.1093/nar/gkv007. 25605792 PMC4402510

[acel70225-bib-0071] Rosset, E. M. , and A. D. Bradshaw . 2016. “SPARC/Osteonectin in Mineralized Tissue.” Matrix Biology: Journal of the International Society for Matrix Biology 52‐54: 78–87. 10.1016/j.matbio.2016.02.001. PMC5327628 26851678

[acel70225-bib-0072] Ryu, S. , S. Sidorov , E. Ravussin , et al. 2022. “The Matricellular Protein SPARC Induces Inflammatory Interferon‐Response in Macrophages During Aging.” Immunity 55, no. 9: 1609–1626.e7. 10.1016/j.immuni.2022.07.007. 35963236 PMC9474643

[acel70225-bib-0073] Scala, M. , M. Mojarrad , S. Riazuddin , et al. 2020. “RSRC1 Loss‐Of‐Function Variants Cause Mild to Moderate Autosomal Recessive Intellectual Disability.” Brain: A Journal of Neurology 143, no. 4: e31. 10.1093/brain/awaa070. 32227164 PMC7174030

[acel70225-bib-0074] Semsei, I. , F. Jeney , and T. Fülöp . 1993. “Effect of Age on the Activity of Ceruloplasmin of Human Blood.” Archives of Gerontology and Geriatrics 17, no. 2: 123–130. 10.1016/0167-4943(93)90044-I. 15374325

[acel70225-bib-0075] Shah, G. N. , B. Ulmasov , A. Waheed , et al. 2005. “Carbonic Anhydrase IV and XIV Knockout Mice: Roles of the Respective Carbonic Anhydrases in Buffering the Extracellular Space in Brain.” Proceedings of the National Academy of Sciences of the United States of America 102, no. 46: 16771–16776. 10.1073/pnas.0508449102. 16260723 PMC1283849

[acel70225-bib-0076] Solesio, M. E. , P. M. Peixoto , L. Debure , et al. 2018. “Carbonic Anhydrase Inhibition Selectively Prevents Amyloid β Neurovascular Mitochondrial Toxicity.” Aging Cell 17, no. 4: e12787. 10.1111/acel.12787. 29873184 PMC6052473

[acel70225-bib-0077] Son, H. G. , O. Altintas , E. J. E. Kim , S. Kwon , and S.‐J. V. Lee . 2019. “Age‐Dependent Changes and Biomarkers of Aging in *Caenorhabditis elegans* .” Aging Cell 18, no. 2: e12853. 10.1111/acel.12853. 30734981 PMC6413654

[acel70225-bib-0078] Sutphin, G. L. , G. Backer , S. Sheehan , et al. 2017. “ *Caenorhabditis elegans* Orthologs of Human Genes Differentially Expressed With Age Are Enriched for Determinants of Longevity.” Aging Cell 16, no. 4: 672–682. 10.1111/acel.12595. 28401650 PMC5506438

[acel70225-bib-0079] Sutphin, G. L. , and M. Kaeberlein . 2009. “Measuring *Caenorhabditis elegans* Life Span on Solid Media.” Journal of Visualized Experiments 27: 1152. 10.3791/1152. PMC2794294 19488025

[acel70225-bib-0080] Sutphin, G. L. , and R. Korstanje . 2016. “Longevity as a Complex Genetic Trait.” In The Handbooks of Aging, Handbook of the Biology of Aging, edited by M. Kaeberlein and G. M. Martin , 4–54. Academic Press.

[acel70225-bib-0081] Tacutu, R. , D. E. Shore , A. Budovsky , et al. 2012. “Prediction of *C. elegans* Longevity Genes by Human and Worm Longevity Networks.” PLoS One 7, no. 10: e48282. 10.1371/journal.pone.0048282. 23144747 PMC3483217

[acel70225-bib-0082] Tan, J. X. , and T. Finkel . 2023. “Lysosomes in Senescence and Aging.” EMBO Reports 24, no. 11: e57265. 10.15252/embr.202357265. 37811693 PMC10626421

[acel70225-bib-0083] Teuscher, A. C. , C. Statzer , A. Goyala , et al. 2024. “Longevity Interventions Modulate Mechanotransduction and Extracellular Matrix Homeostasis in *C. elegans* .” Nature Communications 15, no. 1: 276. 10.1038/s41467-023-44409-2. PMC10766642 38177158

[acel70225-bib-0084] Timmons, L. , and A. Fire . 1998. “Specific Interference by Ingested dsRNA.” Nature 395, no. 6705: 854. 10.1038/27579. 9804418

[acel70225-bib-0085] Toba, H. , and S. Takai . 2024. “Exploring the Roles of SPARC as a Proinflammatory Factor and Its Potential as a Novel Therapeutic Target Against Cardiovascular Disease.” American Journal of Physiology. Heart and Circulatory Physiology 327, no. 5: H1174–H1186. 10.1152/ajpheart.00565.2024. 39269452

[acel70225-bib-0086] van Rossum, G. , and F. L. Drake Jr . 1995. Python Tutorial. Centrum Voor Wiskunde en Informatica.

[acel70225-bib-0087] Volonte, D. , M. Sedorovitz , and F. Galbiati . 2022. “Impaired Cdc20 Signaling Promotes Senescence in Normal Cells and Apoptosis in Non‐Small Cell Lung Cancer Cells.” Journal of Biological Chemistry 298, no. 10: 102405. 10.1016/j.jbc.2022.102405. 35988650 PMC9490043

[acel70225-bib-0088] Wang, B. , and X.‐P. Wang . 2019. “Does Ceruloplasmin Defend Against Neurodegenerative Diseases?” Current Neuropharmacology 17, no. 6: 539–549. 10.2174/1570159X16666180508113025. 29737252 PMC6712297

[acel70225-bib-0089] Wang, L. , J. Zhang , L. Wan , X. Zhou , Z. Wang , and W. Wei . 2015. “Targeting Cdc20 as a Novel Cancer Therapeutic Strategy.” Pharmacology & Therapeutics 151: 141–151. 10.1016/j.pharmthera.2015.04.002. 25850036 PMC4457591

[acel70225-bib-0090] Waskom, M. 2021. “Seaborn: Statistical Data Visualization.” Journal of Open Source Software 6, no. 60: 3021. 10.21105/joss.03021.

[acel70225-bib-0091] Wilkinson, D. S. , R. C. Taylor , and A. Dillin . 2012. “Analysis of Aging in *Caenorhabditis elegans* .” Methods in Cell Biology 107: 353–381. 10.1016/B978-0-12-394620-1.00012-6. 22226530

[acel70225-bib-0092] Williams, G. C. 1957. “Pleiotropy, Natural Selection, and the Evolution of Senescence.” Evolution 11, no. 4: 398–411.

[acel70225-bib-0093] Wirak, G. S. , J. Florman , M. J. Alkema , C. W. Connor , and C. V. Gabel . 2022. “Age‐Associated Changes to Neuronal Dynamics Involve a Disruption of Excitatory/Inhibitory Balance in *C. elegans* .” eLife 11: e72135. 10.7554/eLife.72135. 35703498 PMC9273219

[acel70225-bib-0094] Wu, D. , J. K.‐L. Sun , and K. H.‐M. Chow . 2024. “Neuronal Cell Cycle Reentry Events in the Aging Brain Are More Prevalent in Neurodegeneration and Lead to Cellular Senescence.” PLoS Biology 22, no. 4: e3002559. 10.1371/journal.pbio.3002559. 38652714 PMC11037540

[acel70225-bib-0095] Wu, T. , E. Hu , S. Xu , et al. 2021. “clusterProfiler 4.0: A Universal Enrichment Tool for Interpreting Omics Data.” Innovation 2, no. 3: 100141. 10.1016/j.xinn.2021.100141. 34557778 PMC8454663

[acel70225-bib-0096] Xu, L. , F. Ping , J. Yin , et al. 2013. “Elevated Plasma SPARC Levels Are Associated With Insulin Resistance, Dyslipidemia, and Inflammation in Gestational Diabetes Mellitus.” PLoS One 8, no. 12: e81615. 10.1371/journal.pone.0081615. 24349098 PMC3857203

[acel70225-bib-0097] Xue, H. , B. Xian , D. Dong , et al. 2007. “A Modular Network Model of Aging.” Molecular Systems Biology 3: 147. 10.1038/msb4100189. 18059442 PMC2174624

[acel70225-bib-0098] Yang, Z. , B. V. Alvarez , C. Chakarova , et al. 2005. “Mutant Carbonic Anhydrase 4 Impairs pH Regulation and Causes Retinal Photoreceptor Degeneration.” Human Molecular Genetics 14, no. 2: 255–265. 10.1093/hmg/ddi023. 15563508

[acel70225-bib-0099] Zahn, J. M. , S. Poosala , A. B. Owen , et al. 2007. “AGEMAP: A Gene Expression Database for Aging in Mice.” PLoS Genetics 3, no. 11: e201. 18081424 10.1371/journal.pgen.0030201 PMC2098796

[acel70225-bib-0100] Zečić, A. , and B. P. Braeckman . 2020. “DAF‐16/FoxO in *Caenorhabditis elegans* and Its Role in Metabolic Remodeling.” Cells 9, no. 1: 109. 10.3390/cells9010109. 31906434 PMC7017163

