# Supplementary material for: Six Drivers of Aging Identified Among Genes Differentially Expressed With Age
Source: Aging Cell. 2025 Oct 13;24(11):e70225. doi: 10.1111/acel.70225 (PMC12610947; doi:10.1111/acel.70225)
Supplement: Supplementary file 1 — Table S1: Datasets used in the meta‐analysis of genes differentially expressed during mammalian aging. Table S2: Experimental outcomes of the most consistently age‐downregulated mammalian genes in C. elegans . Table S3: Experimental outcomes of the most consistently age‐upregulated mammalian genes in C. elegans . Figure S1: The number of datasets and DEGs derived from each species and tissue type. Figure S2: Bubble plots showing the expression patterns of the six drivers of aging identified in this study: CASP1, RSRC1, SPARC, CA4, CDC20, and DIRC2. [file ACEL-24-e70225-s001.docx]

**Supplementary Table 1.** Datasets used in the meta-analysis of genes differentially expressed during mammalian aging

| GEO ID | Organism | Platform | Tissue Type | Tissue Subtype | Ages of Subjects |
| --- | --- | --- | --- | --- | --- |
| GSE71868 | Mus musculus | GPL6885 | Immune | Pulmonary CDC11c+ Cells | 6-8wo vs. 10-13mo |
| GSE50821 | Mus musculus | GPL1261 | Muscle | Purified skeletal muscle satellite cells | 2mo vs. 24mo |
| GSE53890 | Homo sapiens | GPL570 | Brain | Frontal cortex | 30-60yo vs. 70-90yo |
| GSE55162 | Mus musculus | GPL1261 | Trachea | NA | 2mo vs. 14mo |
| GSE46646 | Mus musculus | GPL1261 | Liver | NA | 6mo vs. 24mo |
| GSE49543 | Mus musculus | GPL339 | Cochlea | NA | 12mo vs. 30mo |
| GSE38718 | Homo sapiens | GPL570 | Muscle | Skeletal muscle | 19-28yo vs. 65-76yo |
| GSE28422 | Homo sapiens | GPL570 | Muscle | Skeletal muscle (vastus lateralis) | 24±1yo vs. 84±1yo |
| GSE28392 | Homo sapiens | GPL570 | Muscle | Vastus lateralis: type 1 (slow) fibers | 23±2yo vs. 85±1yo |
| GSE25941 | Homo sapiens | GPL570 | Muscle | Skeletal Muscle (Vastus Lateralis) | 25±1yo vs. 78±1yo |
| GSE25905 | Mus musculus | GPL6246 | Fat | Bone marrow adipocytes | 6mo vs. 18mo |
| GSE25905 | Mus musculus | GPL6246 | Fat | Peripheral adipocytes | 6mo vs. 18mo |
| GSE32719 | Homo sapiens | GPL570 | Immune | BM-HSCs | 42-61yo vs. 65-85yo |
| GSE27686 | Mus musculus | GPL1261 | Immune | HSCs | 8mo vs. 24mo |
| GSE24515 | Rattus norvegicus | GPL1355 | Brain | Parietal cortex | 3mo vs. 21mo |
| GSE19677 | Mus musculus | GPL1261 | Brain | Striatum | 12mo vs. 24mo |
| GSE9990 | Rattus norvegicus | GPL341 | Brain | Hippocampus | 12mo vs. 23mo |
| GSE12502 | Canis lupus familiaris | GPL3979 | Muscle | Skeletal muscle: biceps femoris | 8wo vs. 11yo |
| GSE11667 | Mus musculus | GPL1261 | Reproduction | Oocytes | 6-12wo vs. 60-70wo |
| GSE6718^1^ | Rattus norvegicus | GPL1355 | Heart | NA | 4mo vs. 28mo |
| GSE6718^1^ | Rattus norvegicus | GPL1355 | Fat | White adipose tissue | 4mo vs. 28mo |
| GSE8150 | Mus musculus | GPL1261 | Brain | Neocortex | 5mo vs. 30mo |
| GSE8146 | Mus musculus | GPL81 | Heart | NA | 5mo vs. 30mo |
| GSE4270 | Rattus norvegicus | GPL890 | Liver | NA | 12mo vs. 18mo |
| GSE6323 | Mus musculus | GPL339 | Muscle | Skeletal muscle: gastrocnemius | 5mo vs. 25mo |

Datasets were selected according the inclusion and exclusion criteria outlined in Table 1. Samples were broadly categorized into general Tissue Types for grouped analyses, with further details noted in the Tissue Subtype column as available.
^1^As the data series GSE6718 included two sets of samples, each from a distinct tissue type (heart tissue and white adipose tissue), this series was analyzed as two separate datasets and therefore listed in two separate rows.
GEO ID, Gene Expression Omnibus Identifier; NA, Not Applicable; HSCs, hematopoietic stem cells; BM-HSCs, HSCs derived from bone marrow.

**Supplementary Table 2.** Experimental outcomes of the most consistently age-downregulated mammalian genes in *C. elegans*.

| Human DEG | Rank^1^ | Worm Gene | Name | Match^2^ | Ahringer RNAi | Exclusion | Screening Set | Validation Set |
| --- | --- | --- | --- | --- | --- | --- | --- | --- |
| **CA4** | 8 | *K05G3.3* | *cah-3* | 1 | X-7M16 |  | 13%*** (n=71) | 8%*** (n=98) |
| **SIAH2** | 8 | *Y37E11AR.2* | *siah-1* | 4 | IV-8K18 | Failed culture^4^ |  |  |
| **SPARC** | 8 | *C44B12.2* | *ost-1* | 5 | IV-9H03 |  | 23%*** (n=96) | 11%*** (n=143) |
| **UQCR10** | 8 |  |  |  |  | No ortholog |  |  |
| **AR** | 7 |  |  |  |  | No ortholog |  |  |
| **BRD3** | 7 | *Y119C1B.8* | *bet-1* | 5 | I-9E23 |  | 9%** (n=80) | -4%^NS^ (n=97) |
| **BRWD1** | 7 |  |  |  |  | No ortholog |  |  |
| **CDC20** | 7 | *ZK177.6* | *fzy-1* | 1 | II-4O16\|II-4O18^3^ |  | 18%** (n=101) | 15%*** (n=103) |
| **DIRC2** | 7 | *C42C1.8* |  | 1 | IV-6D23 |  | 50%*** (n=92) | 10%*** (n=151) |
| **EIF2D** | 7 | *C25H3.4* |  | 6 | II-10E20\|II-4H08^3^ |  | 2%^NS^ (n=118) |  |
| **EIF4EBP1** | 7 |  |  |  |  | No ortholog |  |  |
| **KIFAP3** | 7 | *F08F8.3* | *kap-1* | 6 | III-3N14 |  | 2%* (n=138) |  |
| **NREP** | 7 |  |  |  |  | No ortholog |  |  |
| **RTN4IP1** | 7 | *F56H1.6* | *rad-8* | 6 | I-9F05 |  | -26%*** (n=77) |  |
| **SMYD1** | 7 | *T22A3.4* | *set-18* | 3 | I-5G16 |  | **-**6% ^NS^ (n=98) |  |
| **VLDLR** | 7 | *T13C2.6* |  | 6 | II-5A06 | Failed culture^4^ |  |  |

Screening lifespan assays were performed for all possible genes, and those producing a statistically significant lifespan extension of 5% or greater were subsequently tested in independent Validation lifespan assays. All experiments were performed using post-developmental RNAi. Results are expressed as mean lifespan extension compared to within-batch GFP controls, with *p*-values calculated using the log-rank test and expressed as: * *p* < 0.05, ** *p* < 0.01, *** *p* < 0.001. ^1^Rank $R$ quantifies how consistently each DEG was downregulated with age, as detailed in the Methods section.
^2^Match quantifies how many orthology programs indicated the ortholog match according to OrthoList2.
^3^Multiple clones were available for some orthologs; the underlined clone was verified and used in RNAi experiments.
^4^Bacterial clones were streaked but failed to grow in standard culture conditions.
DEG, Differentially Expressed Gene.

**Supplementary Table 3.** Experimental outcomes of the most consistently age-upregulated mammalian genes in *C. elegans*.

| Human DEG | Rank^1^ | Worm Gene | Name | Match^2^ | Ahringer RNAi | Exclusion | Screening | Validation |
| --- | --- | --- | --- | --- | --- | --- | --- | --- |
| **EFEMP1** | 11 | *F56H11.1* | *fbl-1* | 1 | IV-4N18\|IV-4P14^3^ |  | 45%*** (n=71) | -4% ^NS^ (n=158) |
| **TMEM176A** | 11 |  |  |  |  | No ortholog |  |  |
| **CP** | 9 |  |  |  |  | No ortholog |  |  |
| **HLA-A** | 9 |  |  |  |  | No ortholog |  |  |
| **ANXA4** | 8 | *T07C4.9* | *nex-2* | 3 | III-5F15\|III-8O24^3^ |  | 6% ^NS^ (n=76) |  |
| **APOE** | 8 |  |  |  |  | No ortholog |  |  |
| **ARAP2** | 8 | *F23H11.4* |  | 3 | III-1G04 |  | -1% ^NS^ (n=119) |  |
| **GCNT2** | 8 | *T15D6.2* | *gly-16* | 4 | I-6A08 |  | -18%*** (n=99) |  |
| **MGST1** | 8 |  |  |  |  | No ortholog |  |  |
| **NPC2** | 8 | *R148.6* | *heh-1* | 1 | III-1P05 | Failed Sanger^5^ |  |  |
| **PRNP** | 8 |  |  |  |  | No ortholog |  |  |
| **PTPRC** | 8 | *F56D1.4* | *clr-1* | 1 | II-4M08 | Failed Sanger^5^ |  |  |
| **SFRP1** | 8 | *Y73B6BL.21* | *sfrp-1* | 4 |  | No clone |  |  |
| **C1QC** | 7 |  |  |  |  | No ortholog |  |  |
| **CASP1** | 7 | *Y47H9C.6* | *csp-3* | 1 | I-5P02 |  | 9%** (n=97) | 9%** (n=101) |
| **CFLAR** | 7 |  |  |  |  | No ortholog |  |  |
| **CTNNA1** | 7 | *R13H4.4* | *hmp-1* | 5 |  | No clone |  |  |
| **CTSS** | 7 | *T03E6.7* | *cpl-1* | 2 | V-11I07 | Failed culture^4^ |  |  |
| **EHD4** | 7 | *W06H8.1* | *rme-1* | 4 | V-14F01 |  | 26%*** (n=100) | 3% ^NS^ (n=133) |
| **FST** | 7 |  |  |  |  | No ortholog |  |  |
| **FYB** | 7 |  |  |  |  | No ortholog |  |  |
| **LYST** | 7 | *T01H10.8* | *lyst-1* | 1 | X-5F04 | Failed culture^4^ |  |  |
| **PJA2** | 7 | *Y54E10BR.3* |  | 1 | I-7N18 |  | -22%*** (n=98) |  |
| **RNF145** | 7 | *Y119C1B.5* |  | 5 | I-9E22 |  | -4% ^NS^ (n=95) |  |
| **RSRC1** | 7 | *C10G11.9* | *spch-2* | 1 | I-3C03 |  | 16%*** (n=98) | 11%*** (n=99) |
| **SERPINB6** | 7 | *F20D6.4* | *srp-7* | 3 | V-14F18 |  | 0% ^NS^ (n=141) |  |
| **SKAP2** | 7 |  |  |  |  | No ortholog |  |  |
| **SLC44A1** | 7 |  |  |  |  | No ortholog |  |  |

Screening lifespan assays were performed for all possible genes, and those producing a statistically significant lifespan extension of 5% or greater were subsequently tested in independent Validation lifespan assays. All experiments were performed using post-developmental RNAi. Results are expressed as mean lifespan extension compared to within-batch GFP controls, with *p*-values calculated using the log-rank test and expressed as: * *p* < 0.05, ** *p* < 0.01, *** *p* < 0.001. ^1^Rank $R$ quantifies how consistently each DEG was downregulated with age, as detailed in the Methods section.
^2^Match quantifies how many orthology programs indicated the ortholog match according to OrthoList2.
^3^Multiple clones were available for some orthologs; the underlined clone was verified and used in RNAi experiments.
^4^Bacterial clones were streaked but failed to grow in standard culture conditions.
^5^Bacterial clones were successfully cultured but were found to be incorrect sequences via Sanger sequencing.
DEG, Differentially Expressed Gene.


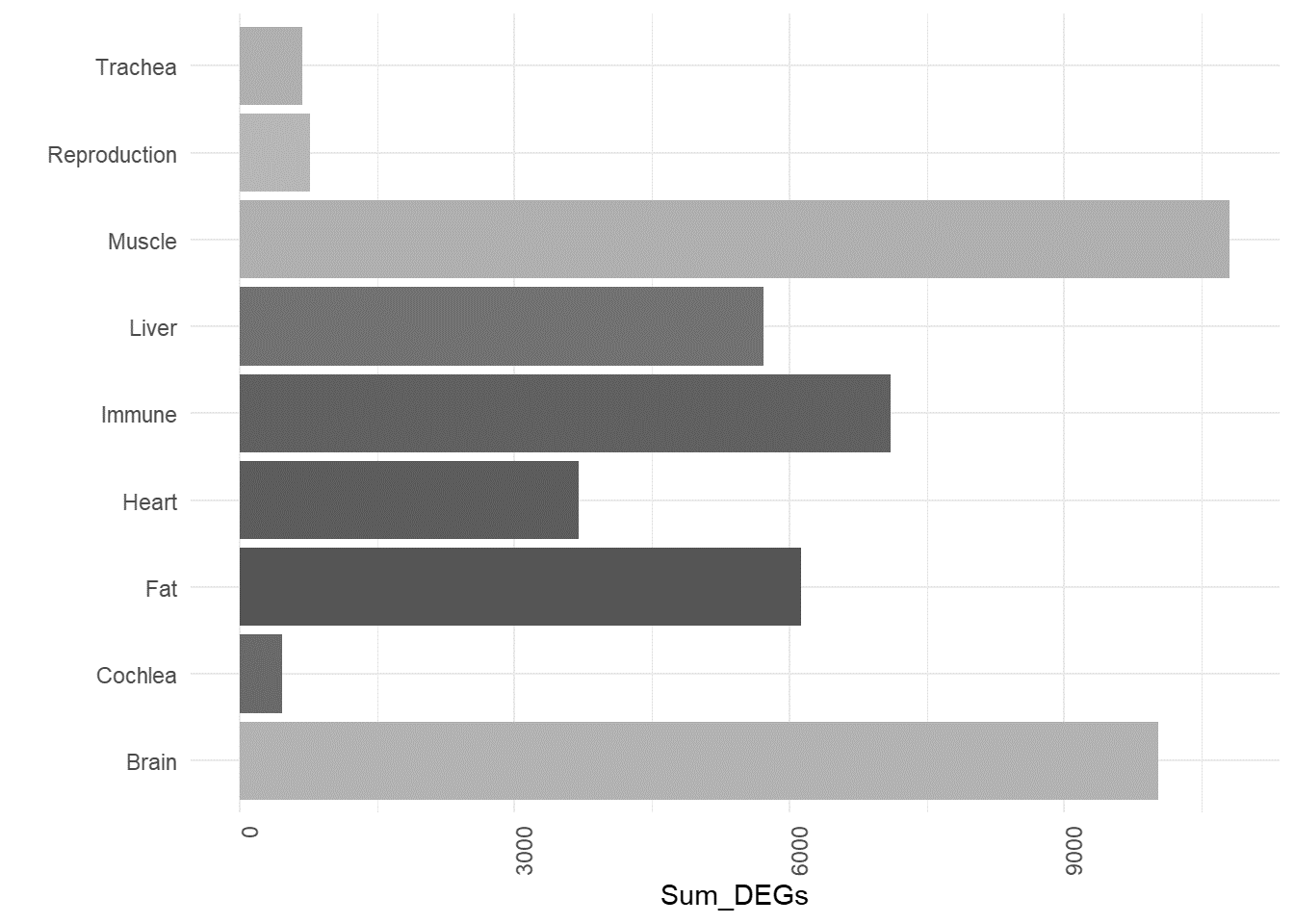

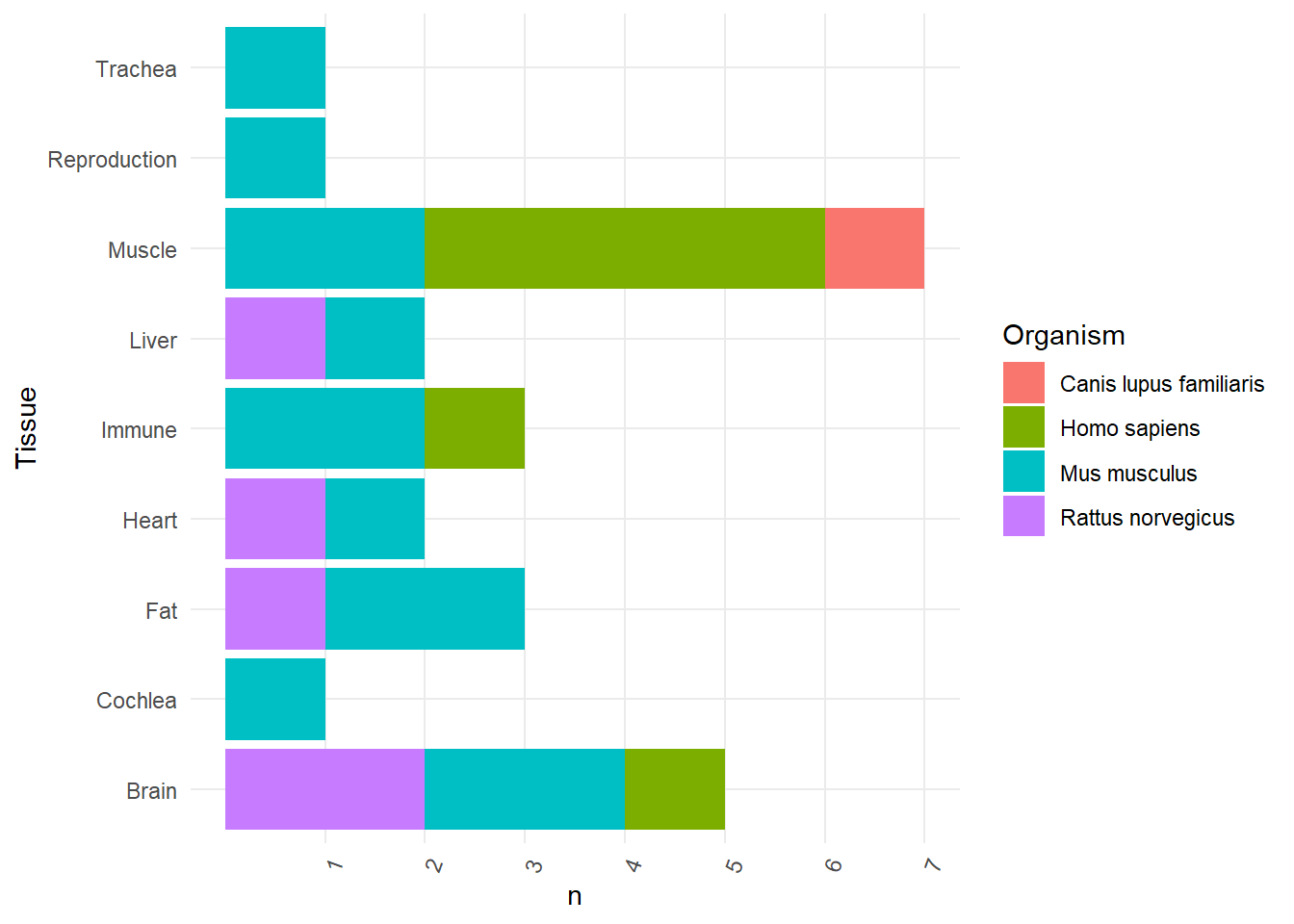

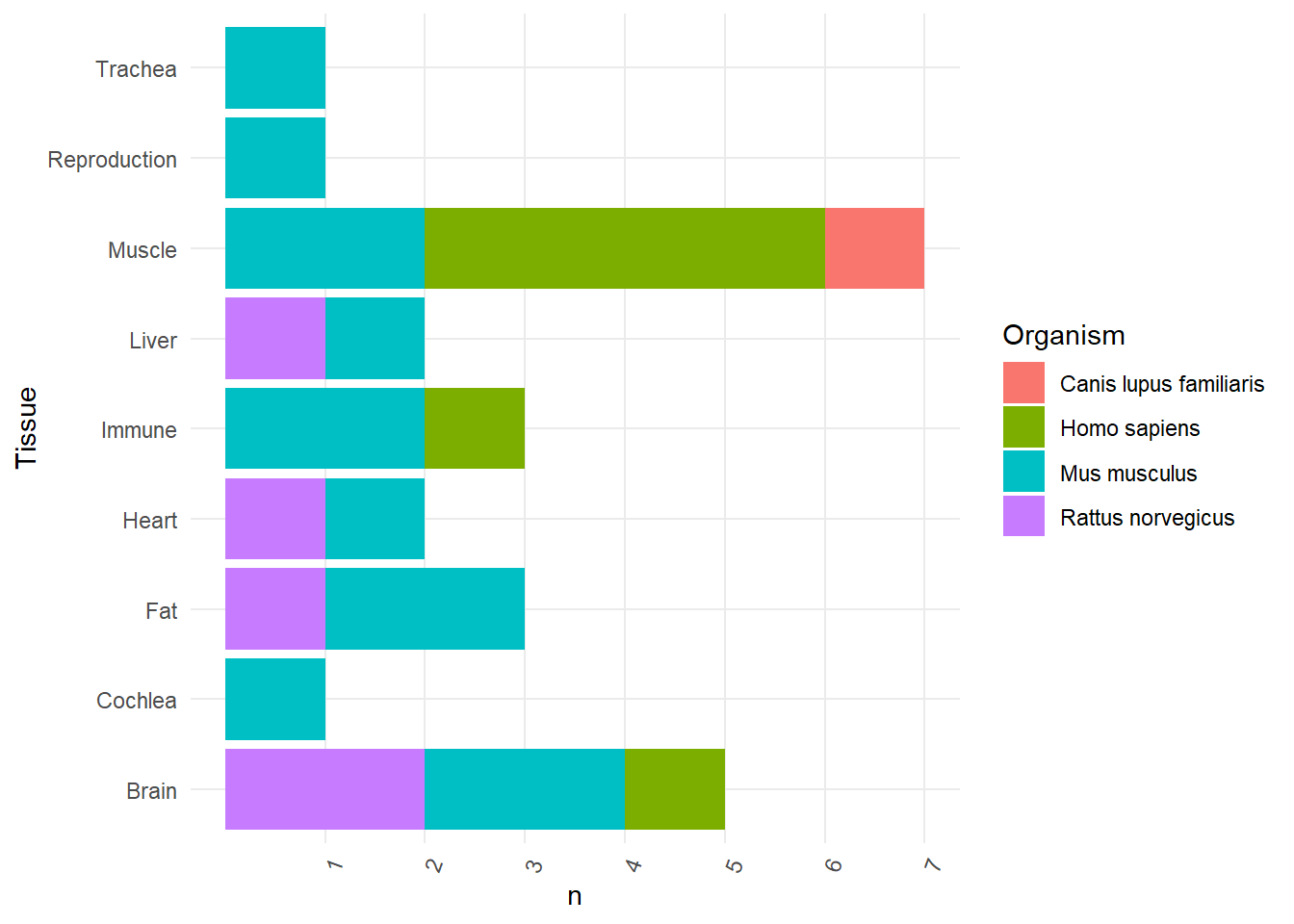


**A**

**B**

**Number of datasets by species and tissue type**

**Number of differentially expressed genes by tissue type**

**Supplementary Figure 1.** The 25 datasets studied were derived from an uneven distribution of species and tissues, and the resulting DEGs were also unevenly distributed across tissue types. (**A**) The total number of datasets (n) derived from each major tissue type, color-coded by the species of origin. (**B**) The sum total number of instances DEGs were identified across all datasets derived from each major tissue type.

**
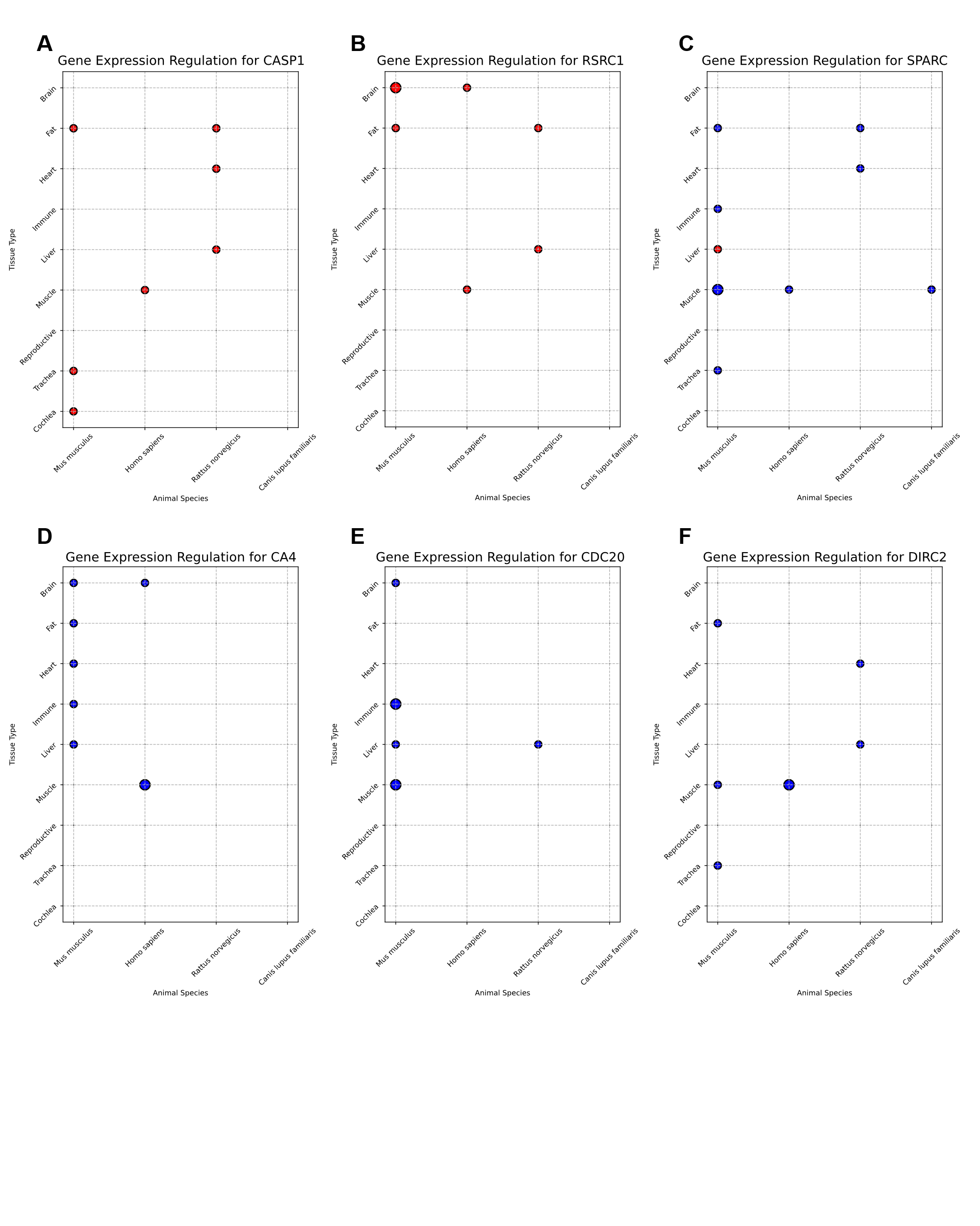
Supplementary Figure 2.** Bubble plots showing the expression patterns of the six drivers of aging identified in this study: CASP1, RSRC1, SPARC, CA4, CDC20, and DIRC2. Bubbles represent significant differential expression between young and old samples: color indicates direction (red for age-upregulated, blue for age-downregulated), and size of the circle indicates the number of datasets exhibiting the displayed trend. Tissue of origin is plotted on each Y-axis (brain, fat, heart, immune, liver, muscle, reproductive, trachea, and cochlea), and species of origin is plotted on each x-axis (mouse, human, rat, dog).
